# Regulation of *Clostridium difficile* Spore Formation by the SpoIIQ and SpoIIIA Proteins

**DOI:** 10.1371/journal.pgen.1005562

**Published:** 2015-10-14

**Authors:** Kelly A. Fimlaid, Owen Jensen, M. Lauren Donnelly, M. Sloan Siegrist, Aimee Shen

**Affiliations:** 1 Department of Microbiology and Molecular Genetics, University of Vermont, Burlington, Vermont, United States of America; 2 Program in Cellular, Molecular & Biomedical Sciences, University of Vermont, Burlington, Vermont, United States of America; 3 Department of Microbiology, University of Massachusetts Amherst, Amherst, Massachusetts, United States of America; Indiana University, UNITED STATES

## Abstract

Sporulation is an ancient developmental process that involves the formation of a highly resistant endospore within a larger mother cell. In the model organism *Bacillus subtilis*, sporulation-specific sigma factors activate compartment-specific transcriptional programs that drive spore morphogenesis. σ^G^ activity in the forespore depends on the formation of a secretion complex, known as the “feeding tube,” that bridges the mother cell and forespore and maintains forespore integrity. Even though these channel components are conserved in all spore formers, recent studies in the major nosocomial pathogen *Clostridium difficile* suggested that these components are dispensable for σ^G^ activity. In this study, we investigated the requirements of the SpoIIQ and SpoIIIA proteins during *C*. *difficile* sporulation. *C*. *difficile spoIIQ*, *spoIIIA*, and *spoIIIAH* mutants exhibited defects in engulfment, tethering of coat to the forespore, and heat-resistant spore formation, even though they activate σ^G^ at wildtype levels. Although the *spoIIQ*, *spoIIIA*, and *spoIIIAH* mutants were defective in engulfment, metabolic labeling studies revealed that they nevertheless actively transformed the peptidoglycan at the leading edge of engulfment. *In vitro* pull-down assays further demonstrated that *C*. *difficile* SpoIIQ directly interacts with SpoIIIAH. Interestingly, mutation of the conserved Walker A ATP binding motif, but not the Walker B ATP hydrolysis motif, disrupted SpoIIIAA function during *C*. *difficile* spore formation. This finding contrasts with *B*. *subtilis*, which requires both Walker A and B motifs for SpoIIIAA function. Taken together, our findings suggest that inhibiting SpoIIQ, SpoIIIAA, or SpoIIIAH function could prevent the formation of infectious *C*. *difficile* spores and thus disease transmission.

## Author Summary

The bacterial spore-forming pathogen *Clostridium difficile* is a leading cause of nosocomial infections in the United States and represents a significant threat to healthcare systems around the world. As an obligate anaerobe, *C*. *difficile* must form spores in order to survive exit from the gastrointestinal tract. Accordingly, spore formation is essential for *C*. *difficile* disease transmission. Since the mechanisms controlling this process remain poorly characterized, we analyzed the importance of highly conserved secretion channel components during *C*. *difficile* sporulation. In the model organism *Bacillus subtilis*, this channel had previously been shown to function as a “feeding tube” that allows the mother cell to nurture the developing forespore and sustain transcription in the forespore. We show here that conserved components of this structure in *C*. *difficile* are dispensable for forespore transcription, although they are important for completing forespore engulfment and retaining the protective spore coat around the forespore, in contrast with *B*. *subtilis*. The results of our study suggest that targeting these conserved proteins could prevent *C*. *difficile* spore formation and thus disease transmission.

## Introduction

A small subset of bacteria can survive adverse environmental conditions by forming a metabolically dormant cell-type known as an endospore (referred to as a “spore” hereafter) [1–3]. Spore formation allows bacteria to survive harsh environmental conditions, such as heat, desiccation, oxygen-rich environments, disinfectants, and antibiotic treatment, since they can “reawaken” when favorable conditions return [1–4]. While spore formation is an ancient and adaptive mechanism for members of the Firmicutes, this developmental process is essential for the survival of many obligate anaerobes that inhabit or transiently live in the gut [5,6].

*Clostridium difficile* is a spore-forming obligate anaerobe that is a leading cause of nosocomial diarrhea and a major threat to healthcare systems around the world [7–10]. When *C*. *difficile* spores are ingested by susceptible hosts, they germinate in the gut and outgrow to form toxin-secreting vegetative cells [7,11,12]. While the toxins produced by *C*. *difficile* are responsible for the disease infection symptoms, spores are essential for this obligate anaerobe to transmit disease [6]. Accordingly, during growth in the gastrointestinal tract, *C*. *difficile* strongly induces sporulation in order to survive exit from the host [6,13]. Spores complicate *C*. *difficile* infection clearance because they are resistant to many disinfectants and inert to antibiotics [4]. As a result, they can persist in the environment for long periods of time and facilitate *C*. *difficile* disease recurrence [12,14,15]. Recurrent *C*. *difficile* infections are particularly problematic because they can lead to severe complications such as pseudomembranous colitis, toxic megacolon, and death [14–16]. However, despite the importance of spores to the pathogenesis of *C*. *difficile*, the molecular mechanisms underlying infectious spore formation remain largely uncharacterized.

Transmission electron microscopy analyses of several spore-forming organisms including *C*. *difficile* have shown that sporulation is defined by a series of morphological events starting with the formation of a polar septum, which generates a larger mother cell and smaller forespore [1–3,17]. The mother cell engulfs the forespore to create a protoplast surrounded by two lipid bilayer membranes suspended within the mother cell cytosol. The germ cell wall between the two membranes serves as the template for the synthesis of a thick protective layer of modified peptidoglycan called the cortex, while a series of protective proteinaceous shells called the spore coat is deposited on the outer forespore membrane [2,18]. Once forespore maturation is complete, the mother cell lyses to liberate a highly resistant spore.

Our knowledge of how these morphological events occur derives primarily from studies of the organism *Bacillus subtilis*. These analyses have revealed that morphological changes during sporulation are coupled to compartment-specific transcriptional changes [1–3]. In particular, the sequential and compartment-specific activation of four conserved sporulation-specific sigma factors, σ^F^, σ^E^, σ^G^, and σ^K^, leads to the activation of transcriptional programs that allow key morphological stages to be completed [1–3,17]. Following asymmetric division, σ^F^ and σ^E^ are activated early in the forespore and mother cell, respectively; following forespore engulfment, σ^G^ and σ^K^ are activated in the forespore and mother cell, respectively. These activation events depend upon coordinated intercompartmental signaling events. σ^F^- and σ^G^-dependent signaling in the forespore activates σ^E^ and σ^K^ in the mother cell, respectively, via regulated intramembrane proteolysis. σ^F^ and σ^E^ control σ^G^ activation in the forespore following engulfment completion by inducing the formation of a channel, also known as the “feeding tube” [19,20]. While the precise composition of this channel has not been determined, “feeding tube” components are thought to physically connect the mother cell to the forespore and transport unknown substrates that are required for σ^G^ activity in the forespore [19–21]. The “feeding tube” also controls forespore integrity [20], since the forespore collapses and eventually lyses in mutants lacking channel components [20,22]. σ^G^ activity may be further regulated by its apparent dependence on engulfment completion [23–25].

Analyses of sporulation-specific sigma factor function in *C*. *difficile* have revealed important differences in the regulatory architecture controlling sporulation [17,26]. While the sigma factors are controlled in a similar compartment-specific manner, σ^E^ activation only partially depends on σ^F^; σ^G^ activation does not require σ^E^; and σ^K^ activation does not depend on σ^G^ [27–29]. Since a *C*. *difficile sigE* mutant, which is stalled at asymmetric division, still activates σ^G^ in the forespore [28], *C*. *difficile* σ^G^ activity does not appear to be coupled to engulfment completion, in contrast with *B*. *subtilis* [23]. In general, activation of *C*. *difficile* sporulation-specific sigma factors appears to depend less on intercompartmental signaling and morphological changes than *B*. *subtilis* [17,26].

Since genome-wide transcriptional profiling has shown that σ^G^ regulon genes are expressed at wildtype levels in a *C*. *difficile sigE* ^*−*^mutant [27,29], the mother cell-to-forespore channel shown to regulate *B*. *subtilis* σ^G^ activity appears to be dispensable for *C*. *difficile* σ^G^ activity, at least at early stages of sporulation [30]. Intriguingly, however, the genes encoding *B*. *subtilis* channel components, *spoIIQ* and the eight gene *spoIIIA* operon, are conserved across all spore-forming bacteria [31,32] and are induced during sporulation in a manner analogous to *B*. *subtilis*, with σ^F^ activating *spoIIQ* transcription and σ^E^ activating *spoIIIA* transcription [27,29]. These observations suggest that the mother cell-to-forespore channel may play important but possibly distinct roles during *C*. *difficile* spore formation relative to *B*. *subtilis* [30].

SpoIIQ has homology to Zn^2+^-dependent M23 peptidases (LytM domain, [33,34]) and forms a multimeric ring in the inner forespore membrane of *B*. *subtilis* [35–38]. The SpoIIIA proteins, SpoIIIAA-SpoIIIAH [39], have homology to secretion system components [19–21,40]. SpoIIIAA appears to function as an ATPase that likely powers the transport of metabolites across the “feeding tube” during *B*. *subtilis* sporulation [20]. SpoIIIAH forms a multimeric ring in the mother cell-derived outer forespore membrane that directly binds the SpoIIQ multimeric ring formed in the inner forespore membrane [34,35,37,38]. The SpoIIQ-SpoIIIAH complex alone can drive “zipper-like” engulfment in sporulating *B*. *subtilis* lacking a cell wall [41]. Based on these observations, this complex has been proposed to function as a Brownian “ratchet” that helps power engulfment. Consistent with this model, a *spoIIQ* mutant fails to complete engulfment [33] when sporulation is induced by nutrient starvation, even though “feeding tube” mutants can complete engulfment when sporulation is induced by resuspension [20].

*C*. *difficile* SpoIIIAA and SpoIIIAH exhibit 57% and 38% similarity, respectively, to their orthologs in *B*. *subtilis* (S1 and S2 Figs), while *C*. *difficile* SpoIIQ (CD0125) exhibits only 28% similarity despite also encoding a C-terminal LytM domain ([32], S3 Fig). In contrast with the degenerate active site of *B*. *subtilis* SpoIIQ [34], *C*. *difficile* SpoIIQ has an intact active site ([30], S3 Fig), suggesting that it may have peptidoglycan endopeptidase activity and thus function differently in *C*. *difficile* relative to *B*. *subtilis*. Furthermore, residues that directly mediate binding between *B*. *subtilis* SpoIIQ and SpoIIIAH are not well conserved in *C*. *difficile* (S1 and S2 Figs), raising the question as to whether these proteins interact in *C*. *difficile*. Indeed, whether SpoIIQ and/or SpoIIIA proteins regulate forespore integrity and/or have additional functions during *C*. *difficile* sporulation remain unknown [30].

To address these questions, we constructed gene disruptions of *C*. *difficile spoIIQ*, *spoIIIAA*, and *spoIIIAH* and determined their effects on spore formation using microscopic and cell biological assays. We also tested whether *C*. *difficile* SpoIIQ and SpoIIIAH interact and whether the predicted ATPase and endopeptidase activities of *C*. *difficile* SpoIIIAA and SpoIIQ are required for spore formation. These analyses revealed that SpoIIQ, SpoIIIAA, and SpoIIIAH regulate multiple stages of *C*. *difficile* spore formation, including engulfment, proper coat localization around the forespore, and maintenance of the forespore.

## Results

### SpoIIQ, SpoIIIAA, and SpoIIIAH are required for spore formation

**Fig 1:**
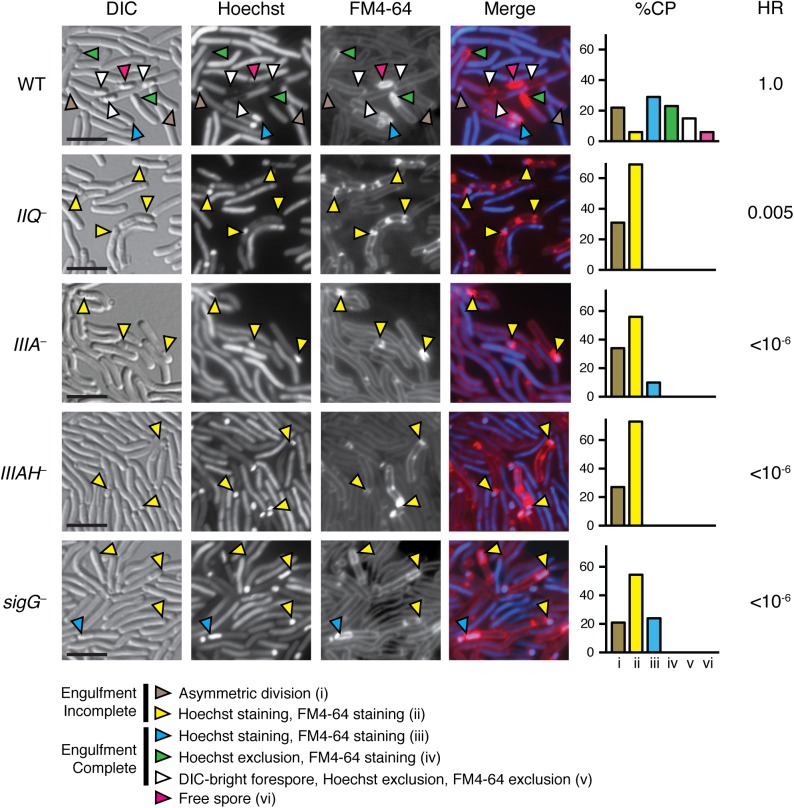
*C*. *difficile spoIIQ*, *spoIIIA*, and *spoIIIAH* mutants are defective in engulfment and mature spore formation. *C*. *difficile* strains wild type (WT), *spoIIQ* ^*−*^(*IIQ* ^*–*^), *spoIIIA* ^*−*^(*IIIA* ^*–*^), *spoIIIAH* ^*−*^(*IIIAH* ^*–*^), and *sigG* ^*−*^were grown on sporulation media for 20 hrs and evaluated by live differential interference contrast (DIC) and fluorescence microscopy. The nucleoid was stained with Hoechst (blue) and membranes were stained with FM4-64 (red). Hoechst appears to be excluded after coat surrounds the forespore [76], while FM4-64 is excluded after membrane fission has occurred at least in *B*. *subtilis* [77]. Brown arrows designate cells at asymmetric division (flat polar septa); yellow arrows designate forespores that have not completed engulfment, although they stain with Hoechst and FM4-64; blue arrows designate cells that have completed engulfment and stain with both Hoechst and FM4-64; green arrows designate forespore compartments that have completed engulfment and exclude Hoechst but stain with FM4-64; white arrows designate forespores that have completed engulfment and exclude Hoechst and FM4-64; pink arrows designate free spores. Free spores were not observed in any of the mutant strains. Cell phenotype percentages (%CP) were determined from analyzing 100 sporulating cells. The efficiency of heat-resistant spore formation (HR) was determined for each strain relative to WT across three biological replicates. Scale bars represent 5 μm.

To first determine if *C*. *difficile* spore development depends on the SpoIIQ and SpoIIIA proteins, we constructed targeted gene disruptions in the σ^F^-regulated *spoIIQ* and σ^E^-regulated *spoIIIAA* and *spoIIIAH* genes using the ClosTron gene knockout system (S4 Fig, [42]). Since targetron insertion into the *spoIIIAA* gene likely causes polar effects on the *spoIIIA* operon, which includes *spoIIIAA-spoIIIAH* (S4 Fig), the *spoIIIAA* mutation will be referred to as a *spoIIIA* mutant from hereon. However, since a second promoter within the *spoIIIA* operon has been shown to drive expression of *spoIIIAG* and *spoIIIAH* ([29], S4 Fig), the *spoIIIA* mutant likely still produces SpoIIIAH. Microscopic analysis of *spoIIQ*, *spoIIIA*, and *spoIIIAH* mutants during sporulation using the membrane dye FM4-64 and the nucleoid dye Hoechst revealed that these mutants are defective in engulfment (Fig 1). The percentage of cells captured at (i) asymmetric division, (ii) pre-engulfment with FM4-64 staining and Hoechst staining, (iii) post engulfment with FM4-64 staining and Hoechst staining, (iv) post engulfment with FM4-64 staining and Hoechst exclusion, (v) post engulfment with DIC-bright, FM4-64 exclusion and Hoechst exclusion, and (vi) free spore was quantified based on analyses of 100 sporulating cells. Whereas uniform staining of FM4-64 around the entire forespore or the presence of DIC-bright spore compartments was observed in wildtype sporulating cells 73% of the time (blue, green, white, and pink arrows), FM4-64 staining of sporulating *spoIIQ* and *spoIIIAH* mutants was restricted to the curved membrane at the mother cell-forespore interface (yellow arrows), and no DIC-bright forespore compartments were observed in these mutants, indicative of an engulfment defect. While the *spoIIQ* and *spoIIIAH* mutants both failed to complete engulfment, the *spoIIIA* and *sigG* mutants completed engulfment 10% and 24% of the time, respectively, although they did not mature to a stage that excluded Hoechst or FM4-64 (Fig 1). Taken together, these results suggest that SpoIIQ and SpoIIIAH are required for *C*. *difficile* forespore engulfment, while the SpoIIIAA-AF complex may be only partially required for engulfment.

Disruption of *C*. *difficile spoIIQ*, *spoIIIA*, and *spoIIIAH* resulted in a significant decrease in heat-resistant spore formation relative to wild type. Interestingly, while the *C*. *difficile spoIIQ* mutant did not show evidence of mature spore formation by fluorescence microscopy, we observed only a 200-fold defect in heat resistance relative to wild type (Fig 1). Since this defect was not as severe as the ~4–6 log defect reported for *B*. *subtilis spoIIQ* mutants [19], we investigated the possibility that the heat-resistant *C*. *difficile spoIIQ* mutant cells might arise from a heritable change by testing the heat resistance of subcultured *spoIIQ* ^*−*^colonies that arose following heat treatment. The same frequency of heat resistance was observed, indicating that the production of heat-resistant *spoIIQ* ^*−*^spores is a stochastic event. In contrast with the *C*. *difficile spoIIQ* mutant, no heat-resistant spores were observed for the *C*. *difficile spoIIIA* ^*−*^and *spoIIIAH* ^*−*^strains within the limits of detection of our assay (<10^−6^, Fig 1). The heat resistance defect of the *C*. *difficile spoIIIA* ^*−*^mutant was similar to the defect reported for a *B*. *subtilis* Δ*spoIIIAA* mutant [20], although the *C*. *difficile spoIIIAH* ^*−*^mutant was at least 4-logs more severe than the defect of a *B*. *subtilis* Δ*spoIIIAH* mutant [20].

**Fig 2:**
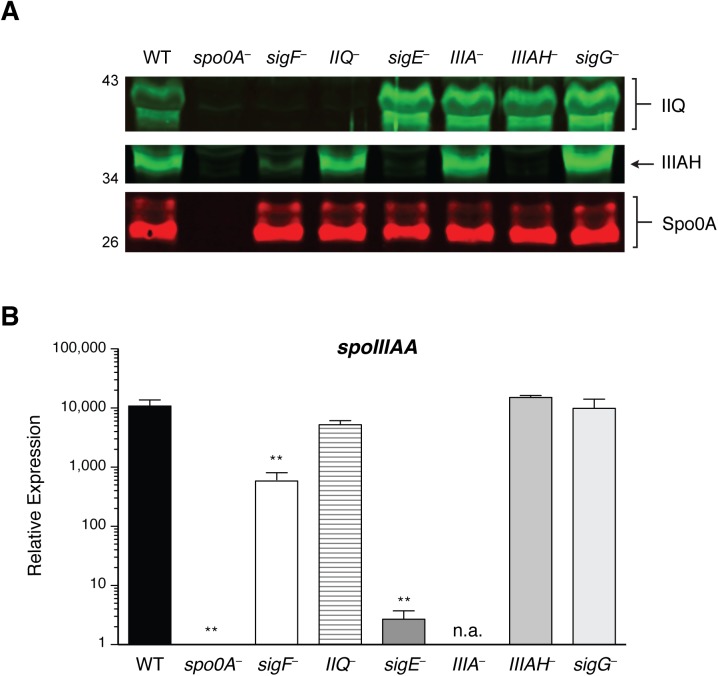
Levels of SpoIIQ and SpoIIIAH in *C*. *difficile* sporulation mutants. (A) Western blot analyses of SpoIIQ and SpoIIIAH levels in cell lysates prepared from WT, *spo0A* ^*–*^, *sigF* ^*–*^, *spoIIQ* ^*−*^(*IIQ* ^*–*^), *sigE* ^*–*^, *spoIIIA* ^*−*^(*IIIA* ^*–*^), *spoIIIAH* ^*−*^(*IIIAH* ^*–*^), and *sigG* ^*−*^strains grown for 17 hr on sporulation media. The anti-Spo0A antibody serves as a control for the extent of sporulation [45]. (B) qRT analysis of *spoIIIAA* transcripts in WT, *spo0A* ^*-*^, *sigE* ^*-*^, *spoIIIA* ^*-*^, and *sigG* ^*-*^ strains grown for 17 hr on sporulation media. Transcript levels were calculated relative to the *spo0A* ^*–*^ strain after normalization to the housekeeping gene *rpoB* using the standard curve method. Data shown represents the averages of three biological replicates. Error bars indicate the standard error of the mean. Statistically significant changes in transcript levels were determined relative to WT and are represented by adjusted p-values determined by a one-way ANOVA and Dunnett’s test. **p ≤ 0.001. n.a. indicates not applicable since the region amplified is downstream of the disrupted *spoIIIAA* gene.

To confirm that the *spoIIQ*, *spoIIIA*, and *spoIIIAH* gene disruptions abrogated protein production, we analyzed sporulating cell lysates prepared from *spoIIQ*, *spoIIIA*, and *spoIIIAH* mutants by Western blotting using antibodies raised against SpoIIQ and SpoIIIAH. As expected, SpoIIQ and SpoIIIAH were not detected in the *spoIIQ* and *spoIIIAH* mutants, respectively, and both proteins were absent in the *spo0A* mutant, which fails to initiate sporulation altogether (Fig 2A). Consistent with *spoIIQ* and *spoIIIAH* regulation by σ^F^ and σ^E^, respectively [27,29], SpoIIQ was absent from the *sigF* mutant, and SpoIIIAH was absent in the *sigE* mutant. SpoIIIAH was nevertheless detected at wildtype levels in the *spoIIIA* mutant, since an internal promoter drives expression of *spoIIIAG-AH* ([29], S4A Fig) similar to the regulation of the *B*. *subtilis spoIIIA* operon [43]. Wildtype levels of SpoIIQ were observed in the *spoIIIAH* mutant, and vice versa, suggesting that loss of the predicted interaction between SpoIIQ and SpoIIIAH did not affect their steady state levels. Wildtype levels of SpoIIQ and SpoIIIAH were also observed in the *sigG* mutant, suggesting that this mutant’s engulfment defect did not result from the absence of these components. The small amount of SpoIIIAH that was detected in a *sigF* mutant (Fig 2A) is consistent with the partial activation of σ^E^ in a *sigF* mutant [27,29].

Since we were unable to generate a working antibody for detecting SpoIIIAA, we measured *spoIIIAA* transcript levels in the same strains. Consistent with the previously reported regulation of *spoIIIAA* by σ^E^ [27,29], statistically significant decreased levels of *spoIIIAA* transcripts were observed in *spo0A*, *sigF*, and *sigE* mutants relative to wild type (Fig 2B, p < 0.01). *spoIIIAA* transcript levels were unaffected in the *spoIIQ*, *spoIIIAH*, and *sigG* mutants (Fig 2B), indicating that loss of SpoIIQ or SpoIIIAH does not alter *spoIIIAA* expression. *spoIIIAA* transcripts could not be accurately measured in the *spoIIIA* mutant, since the amplification product is downstream of the targetron insertion.

### Plasmid complementation rescues the sporulation defects of *spoIIQ*, *spoIIIA*, and *spoIIIAH* mutants

To validate that the observed mutant phenotypes were due to the targeted insertions, we attempted to complement the mutant strains with a wildtype copy of the disrupted gene(s) expressed from their native promoter using the pMTL83151 multicopy plasmid [44]. The *spoIIIA* mutant was complemented with the full *spoIIIA* operon, and the *spoIIIAH* mutant was complemented with either the full *spoIIIA* operon, or the *spoIIIAH* gene alone (S5 Fig). The *spoIIQ* and *spoIIIA* complementation constructs all restored production of heat-resistant, DIC-bright spores to their respective mutant backgrounds (S5 Fig). While complementation of the *spoIIIAH* mutant with either the *spoIIIA* operon or *spoIIIAH* gene under the control of the *spoIIIA* promoter restored heat-resistant spore production, the *spoIIIA* operon conferred ~8-fold higher heat-resistance to the *spoIIIAH* mutant relative to complementation with the *spoIIIAH* gene alone. Western blot analysis revealed that SpoIIIAH levels were elevated in the *spoIIIA* operon complementation strain relative to *spoIIIAH* complementation strain and wildtype carrying empty vector (S6 Fig). Complementation of *spoIIQ* ^*−*^resulted in ~4-fold greater heat-resistant spore formation than wildtype carrying empty vector. Western blot analysis indicated that SpoIIQ levels were slightly elevated in the *spoIIQ* complementation strain relative to wildtype carrying empty vector (S6 Fig).

Fluorescence microscopy analyses of the *spoIIQ*, *spoIIIA*, and *spoIIIAH* strains carrying empty vector confirmed that the majority of mutant cells failed to complete engulfment (S5 Fig, yellow arrows). On rare occasions, we observed that the *spoIIIA* mutant carrying empty vector completed engulfment (S5 Fig, blue arrow), similar to our observations with the *spoIIIA* mutant alone (Fig 1). Regardless, these results indicate that the gene disruptions in the *spoIIQ*, *spoIIIAA* and *spoIIIAH* genes are responsible for the observed engulfment and heat-resistance defects.

### Engulfment defects in the *spoIIQ*, *spoIIIA*, and *spoIIIAH* mutants

**Fig 3:**
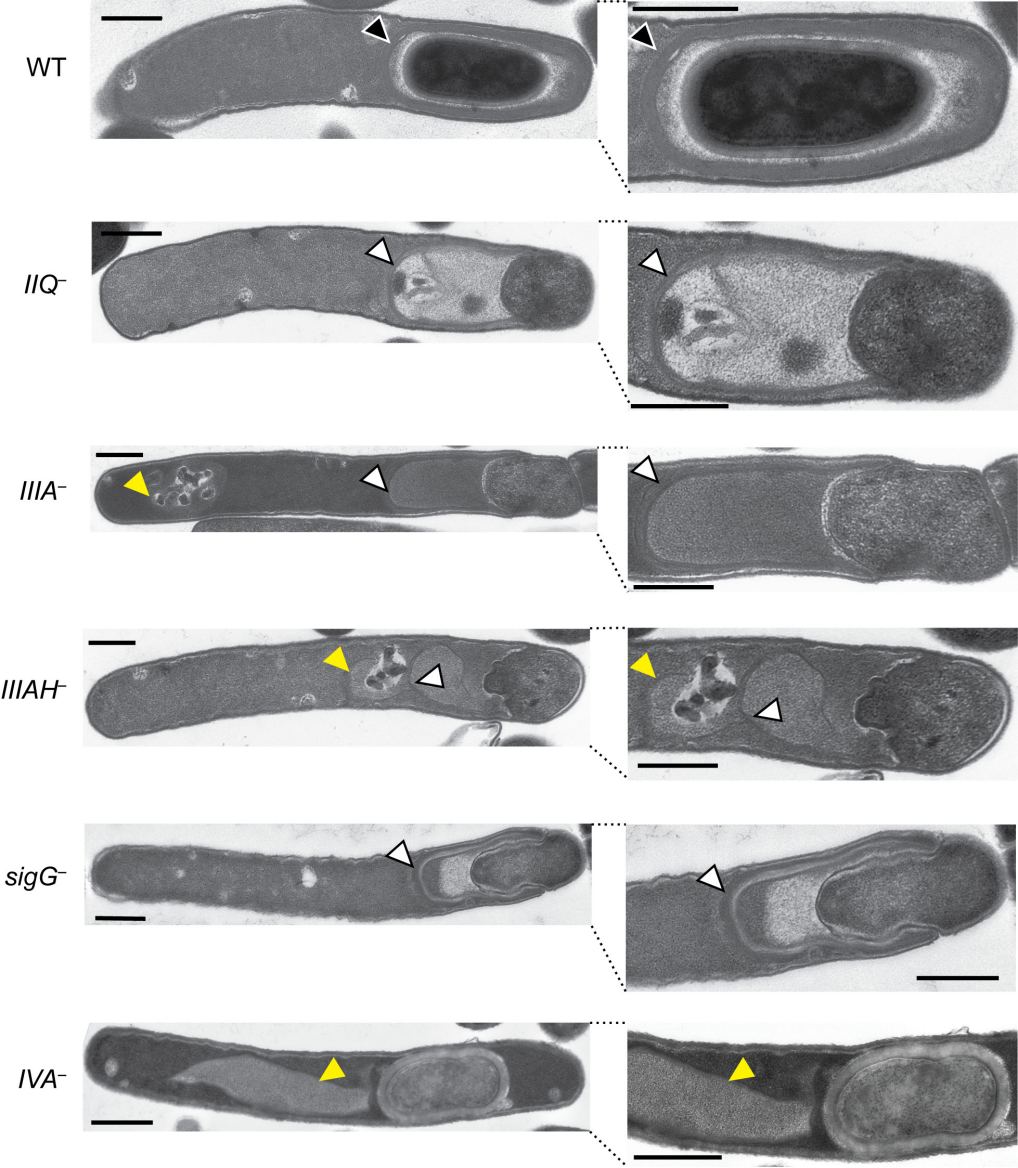
Morphological defects of *spoIIQ*, *spoIIIA*, and *spoIIIAH* mutants. Transmission electron microscopy (TEM) of WT, *spoIIQ* ^*−*^(*IIQ* ^*–*^), *spoIIIA* ^***−***^(*IIIA* ^*–*^), *spoIIIAH* ^***−***^(*IIIAH* ^*–*^), *sigG* ^*–*^, and *spoIVA* ^*−*^(*IVA* ^*–*^) strains grown for 24 hrs on sporulation media. The forespore region of these strains is shown on the right. Black arrows indicate regions that resemble coat layers surrounding the forespore. White arrows indicate coat that appears anchored to the leading edge of the engulfing membrane but is not intimately associated with the mother cell-forespore interface. Yellow arrows highlight coat that appears to be mislocalized away from the forespore region to the mother cell cytosol. Scale bars represent 500 nm.

To gain further insight into the nature of the engulfment defect in the *spoIIQ*, *spoIIIA*, and *spoIIIAH* mutants, we analyzed each mutant using transmission electron microscopy (TEM). We failed to observe engulfment of *spoIIQ* and *spoIIIAH* mutants based on analyses of over 50 cells that had progressed beyond asymmetric division for each mutant, with engulfment being defined as the mother cell-derived membrane surrounding the entire forespore (Fig 3). The *spoIIIA* mutant was observed to complete engulfment in ~20% of cells analyzed by TEM, even though this mutant failed to produce heat-resistant spores (Fig 1). In contrast, none of the *spoIIQ* and *spoIIIAH* mutant cells strains completed engulfment. However, since heat-resistant *spoIIQ* ^*−*^spores could be detected at a frequency of 1 in 200 (Fig 1), we extensively analyzed the TEM grids and identified a single *spoIIQ* ^*−*^cell that had completed engulfment (S7A Fig). Taken together, these results confirm the live cell microscopy analyses (Fig 1): loss of SpoIIQ and SpoIIIAH causes a severe defect in forespore engulfment, while the apparent loss of SpoIIIAA-AF in the *spoIIIA* mutant still permits engulfment in 10–20% of cells.

### *spoIIQ*, *spoIIIA*, and *spoIIIAH* mutants exhibit defects in adhering coat to the forespore and maintaining forespore integrity

In addition to the engulfment defects observed in the *spoIIQ*, *spoIIIA*, and *spoIIIAH* mutants by TEM, a second “compartment” was often observed to extend from the forespore of the *spoIIQ*, *spoIIIA*, and *spoIIIAH* mutants (Fig 3, white arrows). Closer inspection of these extensions revealed multiple striated lines that were consistent with coat. The mutant coat-like structures appeared to anchor to the leading edge of the engulfing membrane but were not adhered to the mother cell-forespore interface in the majority of cells with engulfment defects. Coat-like structures were present 100% of the time in *spoIIQ* ^*–*^, *spoIIIA* ^*–*^, and *spoIIIAH* ^*−*^strains that had begun engulfment; these structures appeared anchored to the leading edge of the engulfing membrane 94%, 96%, and 66% of the time in *spoIIQ* ^*–*^, *spoIIIA* ^*–*^, and *spoIIIAH* ^*−*^strains, respectively. A similar phenotype was observed in the *sigG* ^*−*^strain (Fig 3, [27]). In some instances, the coat-like structures were not associated with the forespore at all and were instead mislocalized to the mother cell cytosol, similar to the previously described coat mislocalization phenotype of a *spoIVA* mutant ([45], Fig 3, yellow arrows). In particular, mislocalized cytosolic coat was observed with high frequency in the *spoIIIAH* mutant (51%), regardless of whether the coat was anchored to the leading edge. For the *spoIIQ* ^*–*^, *spoIIIA* ^*–*^, and *spoIIIAH* ^*−*^cells that had coat anchored to the leading edge of the engulfing membrane, 98%, 89%, and 94%, respectively, did not have coat intimately associated with the forespore interface (Fig 3, white arrows). Notably, the rare *spoIIIA* and *spoIIQ* mutants that completed engulfment had visible coat surrounding the forespore compartment by TEM (S7A Fig, black arrows). Furthermore, in wildtype cells, coat was only observed after engulfment was complete.

**Fig 4:**
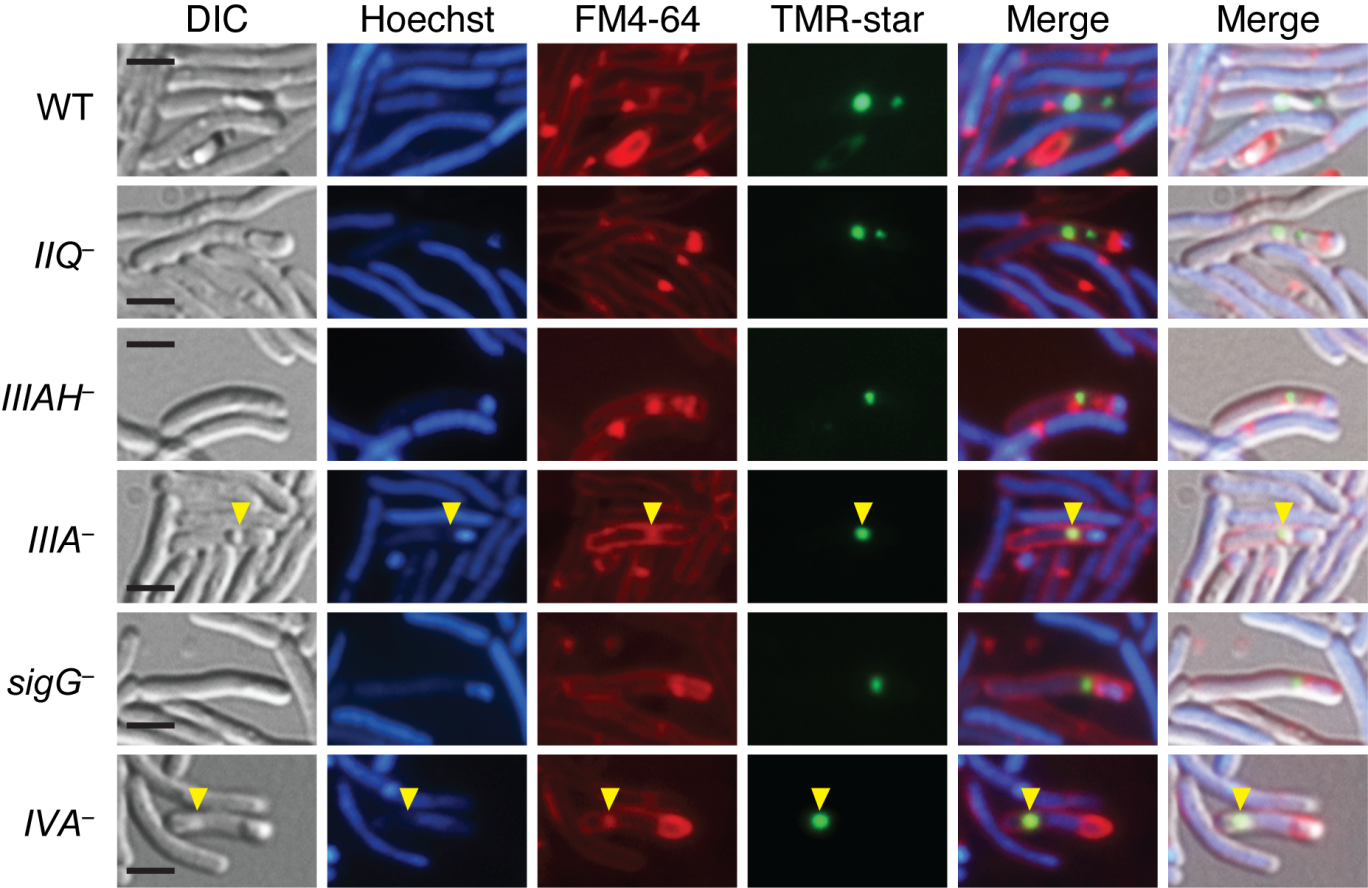
Coat mislocalization in the absence of SpoIIQ and SpoIIIA proteins. DIC and fluorescence microscopy of WT, *spoIIQ* ^*−*^(*IIQ* ^*–*^), *spoIIIA* ^***−***^(*IIIA* ^*–*^), *spoIIIAH* ^***−***^(*IIIAH* ^*–*^), *sigG* ^*–*^, and *spoIVA* ^*−*^(*IVA* ^*–*^) strains producing CotE-SNAP fusions. Cells were collected after 24 hrs on sporulation media and labeled with the SNAP substrate TMR-Star (green), the lipophilic dye FM4-64 (red), and the Hoechst nucleoid dye (blue). Yellow arrows indicate where the SNAP signal (green) overlaps with FM4-64 staining (red). Scale bars represent 2 μm.

To confirm that the coat-like assemblages observed in the *spoIIQ*, *spoIIIA*, *spoIIIAH*, and *sigG* mutants were indeed coat, we analyzed the localization of a known coat protein in these mutant backgrounds. In particular, we correlated the localization of the previously reported surface-exposed coat protein CotE fused to a SNAP imaging tag [28] with FM4-64 and Hoechst staining. The CotE-SNAP protein fusion was detected concentrated at both poles of the developing forespore in wild type, with a weaker signal surrounding the forespore (Fig 4) similar to the previously reported localization of this protein fusion around the forespore [28]. Faint CotE-SNAP staining was observed around free spores of wild type, consistent with the surface localization of CotE [46,47]. In contrast, in the *spoIIQ*, *spoIIIA*, and *spoIIIAH* mutants, CotE-SNAP signal was frequently offset from FM4-64 staining of the forespore membrane. The CotE-SNAP signal was also observed mislocalized to the mother cell cytosol in these mutants (Fig 4), similar to the displacement of the CotE-SNAP signal to the mother cell cytosol of the *spoIVA* mutant, which has previously been shown to mislocalize coat [45]. While the FM4-64 readily stained forespore membranes, it also appeared to associate with mislocalized coat in the *spoIVA*, *spoIIQ*, *spoIIIA*, and *spoIIIAH* mutants (Fig 4, yellow arrows), making it difficult to assess by light microscopy whether CotE-SNAP was adhered to the forespore membrane. Nevertheless, combined with our TEM data, the CotE-SNAP localization experiments strongly suggest that coat detaches from the forespore and/or completely mislocalizes to the mother cell cytosol in the absence of SpoIIQ and SpoIIIA proteins.

While the dominant phenotypes observed by TEM for *spoIIQ*, *spoIIIA*, and *spoIIIAH* mutants are shown in Fig 3, 13%, 14%, and 27% of *spoIIQ*, *spoIIIA*, and *spoIIIAH* mutant cells, respectively, harbored forespores that were undergoing forespore collapse. In particular, large invaginations of the forespore membrane were observed in these mutants (S7B Fig, blue arrows), similar to the phenotypes previously described for *B*. *subtilis* mutants lacking SpoIIQ or SpoIIIA complex components [20]. These results indicate that these proteins in *C*. *difficile* are also required to maintain forespore integrity, similar to *B*. *subtilis* [20].

### *C*. *difficile* σ^G^ activity does not depend on SpoIIQ, SpoIIIAA, and SpoIIIAH

In addition to maintaining forespore integrity in *B*. *subtilis*, the feeding tube is required to sustain transcription in the forespore [19] and thus is necessary for σ^G^ activity [19,20,25,40]. However, previous transcriptional analyses in *C*. *difficile* suggested that the “feeding tube” components were dispensable for σ^G^ activity, since a *C*. *difficile* σ^G^-dependent transcriptional reporter is produced in the forespore of a *sigE* mutant [28], and the σ^G^ regulon is expressed at wildtype levels in the *sigE* mutant [27,29]. To test whether *C*. *difficile spoIIQ*, *spoIIIA*, and *spoIIIAH* are required for σ^G^ activity, we measured σ^G^-dependent transcript levels in wild type, *spoIIQ*, *spoIIIA* and sporulation sigma factor mutants using quantitative RT-PCR. As expected, no statistically significant difference in σ^G^-dependent transcripts *spoVT*, *spoVAD*, and *CD1430* [27,29], were observed in the feeding tube mutants (S8 Fig). Consistent with the dependence of σ^G^ activity on Spo0A and σ^F^, *spoVT* was significantly decreased in *spo0A*, *sigF*, and *sigG* mutants (p < 0.0005), *CD1430* was significantly decreased in *spo0A*, *sigF*, and *sigG* mutants (p < 0.05) and *spoVAD* was significantly decreased in *spo0A*, *sigF*, and *sigG* mutants (p < 0.01, 0.01, and 0.05, respectively).

**Fig 5:**
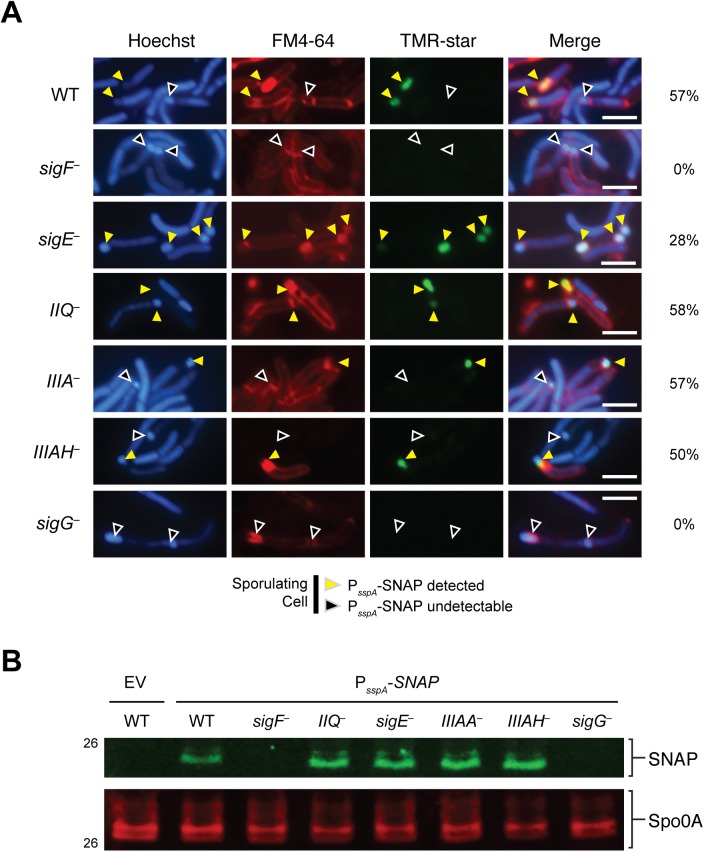
σ^G^ activity is localized to the forespore in *spoIIQ*, *spoIIIAA*, and *spoIIIAH* mutants. (A) Fluorescence microscopy analyses of the indicated strains carrying the σ^G^-dependent P_*sspA*_-SNAP transcriptional reporter. Cells were collected after growth on sporulation media for 22 hr and stained with TMR-Star to monitor σ^G^ activity (green), the lipophilic dye FM4-64 (red), and Hoechst nucleoid dye (blue). Yellow arrows denote forespores where σ^G^-activity is detectable by TMR-Star staining. Black arrows mark sporulating cells where σ^G^ activity was not observed. The percentage of cells for each strain producing visible SNAP for each strain is shown (at least 50 sporulating cells per strain were counted). (B) Western blot analyses of strains carrying the σ^G^-dependent P_*sspA*_ *-*SNAP transcriptional reporter using an anti-SNAP antibody. The anti-Spo0A antibody serves as a control for the extent of sporulation [45]. Scale bars represent 3 μm.

Since it is possible that the wildtype levels of σ^G^ activity detected in *spoIIQ* and *spoIIIA* mutants by qRT-PCR may derive from improper σ^G^ activation in the mother cell, we used the σ^G^-dependent SNAP-tag transcriptional reporter to visualize σ^G^ activation in *spoIIQ* and *spoIIIA* mutants. The promoter region of the σ^G^-dependent *sspA* gene previously described by Pereira *et al*. [28] was fused to a codon-optimized *SNAP* gene and conjugated into wildtype, *sigF* ^–^, *spoIIQ* ^–^, *sigE* ^–^, *spoIIIA* ^–^, *spoIIIAH* ^–^, and *sigG* ^−^strains. Similar to the previous reports [28], SNAP labeling with the TMR-Star substrate (i.e. σ^G^ activity) was restricted to the forespore of cells that had completed asymmetric division or engulfment (Fig 5A). No SNAP signal was detectable in either the *sigF* or *sigG* mutants (Fig 5A, black arrows), as expected. In contrast, σ^G^-dependent SNAP labeling was detectable in the forespores of cells undergoing sporulation in the *spoIIQ* ^–^, *sigE* ^–^, *spoIIIA* ^–^, and *spoIIIAH* ^−^strains (Fig 5A, yellow arrows). The prevalence of σ^G^-dependent transcription in cells undergoing sporulation (based on the presence of an asymmetric septum, engulfment initiated, or engulfment completed phenotype) was determined by counting the number of cells exhibiting SNAP labeling. The σ^G^-dependent transcriptional reporter was produced in the forespore of *spoIIQ* ^–^, *spoIIIA* ^–^, and *spoIIIAH* ^−^cells 58%, 57%, and 50%, respectively, of sporulating cells, which was similar to the frequency observed in wild type (57%, Fig 5A). The SNAP signal was observed in the forespore of the *sigE* mutant in 28% of sporulating cells. Western blot analyses confirmed that wildtype levels of SNAP protein were observed in *spoIIQ*, *sigE*, *spoIIIA*, and *spoIIIAH* mutants (Fig 5B). Taken together, these results demonstrate that the *C*. *difficile* SpoIIQ and SpoIIIA components are dispensable for maintaining transcription in the forespore, in contrast with *B*. *subtilis* [19].

### SpoIIQ and SpoIIIAH interact

**Fig 6:**
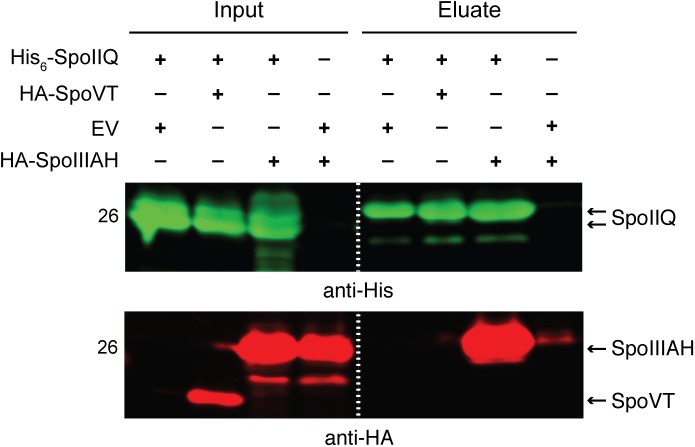
*C*. *difficile* SpoIIQ and SpoIIIAH directly interact *in vitro*. Western blot analyses of co-affinity purifications of His_6_-tagged SpoIIQ with either empty vector (EV), HA-SpoVT, and HA-SpoIIIAH. The indicated constructs were produced in *E*. *coli* upon induction with IPTG. Input represents the soluble fraction of cell lysates prepared from the co-expression strains prior to Ni^2+^-affinity purification, while eluate represents the fractions eluted from the Ni^2+^-affinity beads following incubation with the soluble fraction. Dual western blot detection was performed using anti-HA (red) and anti-His (green).

Since these analyses indicated that the *C*. *difficile* SpoIIQ and SpoIIIA proteins regulate different cellular processes during sporulation than in *B*. *subtilis*, namely σ^G^ activity and forespore engulfment, we next sought to investigate how these components regulated these processes. We first tested whether the *C*. *difficile* feeding tube components assemble into a complex as has been shown in *B*. *subtilis* [34], since the interaction between SpoIIQ and SpoIIIAH is necessary for feeding tube function in *B*. *subtilis* [35]. Using a co-affinity purification assay, we determined whether *C*. *difficile* SpoIIQ and SpoIIIAH directly interact through their extracellular domains. To this end, we co-expressed His_6_-tagged SpoIIQ and HA-tagged SpoIIIAH, both lacking their transmembrane domains, in *E*. *coli*. Affinity purification of His_6_-tagged SpoIIQ resulted in the co-purification of HA-tagged SpoIIIAH, whereas HA-tagged SpoVT, which was used as a specificity control, did not co-purify with His_6_-tagged SpoIIQ when co-expressed (Fig 6). Thus, despite the low degree of sequence homology between *B*. *subtilis* and *C*. *difficile* SpoIIQ orthologs (S3 Fig), *C*. *difficile* SpoIIQ and SpoIIIAH directly interact *in vitro* through their extracellular domains, consistent with the hypothesis that these proteins form a complex that bridges the intercompartmental space between the mother cell and forespore [34,41].

### Mutation of the SpoIIQ LytM catalytic triad does not strongly impact sporulation

Based on these findings, we next tested whether the predicted catalytic activities of SpoIIQ and SpoIIIAA were required for their function. The LytM domain of *C*. *difficile* SpoIIQ carries an intact catalytic triad consisting of two conserved motifs: HxxxD and HxH [30]. These motifs coordinate a metal ion (commonly zinc) that is essential for endopeptidase activity, which degrades the peptide linkages that crosslink the glycan strands of peptidoglycan [48]. To determine if *C*. *difficile* SpoIIQ’s endopeptidase activity is necessary for sporulation, we complemented the *spoIIQ* mutant with a *spoIIQ* variant encoding a histidine 120 to alanine mutation (*spoIIQ* ^*–*^ */*H120A, S3 Fig), which should inactive its predicted endopeptidase activity. Analysis of this strain in the heat resistance assay indicated that the H120A mutation caused an ~50% reduction in heat-resistant spore formation relative to wildtype carrying empty vector (S9 Fig). TEM analyses revealed that 52% of *spoIIQ* ^*–*^ */*H120A cells completed engulfment compared to 88% of the wildtype complementation strain (*spoIIQ* ^*–*^/*spoIIQ*) and 96% of wildtype carrying empty vector (S9 Fig). These results indicate that the endopeptidase activity of SpoIIQ plays a minor role in regulating *C*. *difficile* sporulation under the conditions tested.

### The Walker A motif of SpoIIIAA is important for *C*. *difficile* sporulation

**Fig 7:**
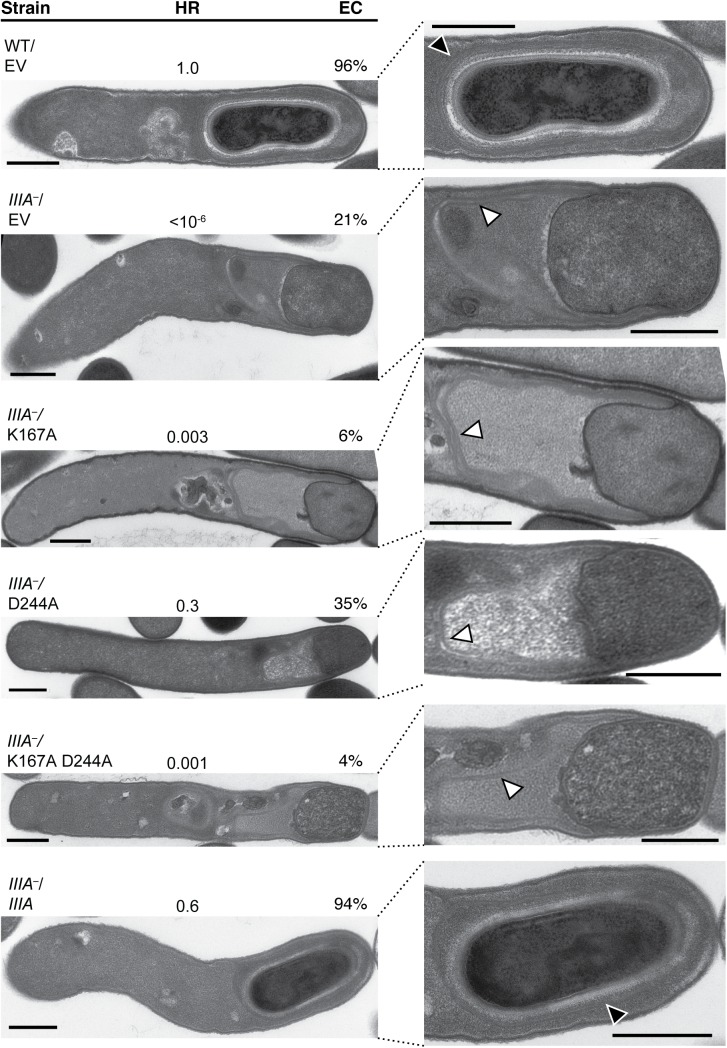
Mutation of the Walker A motif of SpoIIIAA leads to defects in spore formation. TEM analyses of wildtype carrying empty vector (WT/EV) and *spoIIIA* ^*−*^(*IIIA* ^*–*^) strains carrying empty vector (EV), or *spoIIIA* complementation constructs encoding the wildtype operon (*IIIA*), a K167A Walker A mutation (K167A), D244A Walker B mutation (D244A), and K167A/D244A double mutation (K167A/D244A). The forespore region of these strains is shown on the right. Black arrows indicate regions that resemble coat layers surrounding the forespore. White arrows indicate coat that appears anchored to the leading edge of the engulfing membrane but is not intimately associated with the mother cell-forespore interface. Scale bars represent 500 nm. The efficiency of heat-resistant (HR) spore formation was determined for each strain relative to WT for at least three biological replicates. Engulfment complete (EC) cells designates the number of cells in the population that completed engulfment out of at least 50 sporulating cells that had initiated engulfment or progressed beyond this stage.

SpoIIIAA is predicted to function as an ATPase, since strains carrying mutations of conserved residues in the Walker A and B boxes (S1 Fig, [49]) in *B*. *subtilis* resemble a Δ*spoIIIAA* mutant [20]. Disruption of the Walker A motif typically prevents ATP binding [50], while disruption of the Walker B motif typically prevents ATP hydrolysis without affecting ATP binding [51,52]. To determine whether the ATPase activity of *C*. *difficile* SpoIIIAA is also required for spore formation, we constructed complementation strains encoding SpoIIIAA carrying a Walker A lysine mutation, K167A, a Walker B aspartate mutation, D244A, and a Walker A/Walker B double mutation K167A/D244A (S1 Fig), and tested their ability to complement the heat resistance defect of a *spoIIIA* mutant. The K167A mutant, expressed from a *spoIIIA* operon complementation plasmid exhibited ~300-fold defect relative to wildtype carrying empty vector (Fig 7). Interestingly, only 6% of sporulating K167A cells completed engulfment when analyzed by TEM compared to 20% of the parent *spoIIIA* ^*−*^strain carrying empty vector (Fig 7), and similar results were observed by FM4-64 and Hoechst staining (S10 Fig). The D244A mutant exhibited close to wildtype levels of heat resistance (~3-fold decrease), consistent with its ability to complete engulfment 35% of the time (Fig 7). The K167A/D244A double mutant resembled the K167A single mutant in exhibiting a three-log decrease in heat resistance and ~4% engulfment efficiency relative to wild type. In contrast, the wildtype complementation strain (*spoIIIA* ^*–*^/*spoIIIA*) exhibited wildtype levels of heat resistance and engulfment completion (Fig 7).

Since we could not test whether the K167A, D244A, or K167A/D244A mutation(s) affected SpoIIIAA protein levels due to the absence of a working antibody, we compared *spoIIIAA* transcript levels in the K167A mutant, whose heat resistance and engulfment defect was more severe than the D244A mutant and equivalent to the double mutant (Fig 7), relative to wildtype carrying empty vector and the *spoIIIA* complementation strain. These analyses indicated that *spoIIIAA* transcript levels in the K167A complementation strain were similar to the *spoIIIA* complementation strain and wildtype carrying empty vector (S6B Fig). Since the Walker A K167A mutation was considerably more severe than the Walker B D244A mutation, our results suggest that SpoIIIAA function likely depends on its ability to bind, but not necessarily hydrolyze, ATP. Given that *B*. *subtilis* SpoIIIAA function completely depends on the presence of intact Walker A and Walker B boxes [20], *C*. *difficile* SpoIIIAA appears to have differential requirements for its function relative to *B*. *subtilis*.

### *spoIIQ*, *spoIIIA*, and *spoIIIAH* mutants actively transform peptidoglycan around the forespore despite their engulfment defects

The engulfment defects of the *C*. *difficile spoIIQ*, *spoIIIA*, and *spoIIIAH* mutants prompted us to investigate the mechanisms underlying this engulfment defect. In *B*. *subtilis*, peptidoglycan hydrolase enzymes that degrade the peptidoglycan layer between the mother cell and forespore drive engulfment [53–55]. Subsequent transformations of this peptidoglycan layer, which involve both the making and breaking of peptide and glycan bonds, essentially “cut” the forespore free of the mother cell until engulfment is complete [56]. Since transpeptidases and/or ligases can incorporate D-alanine into the stem peptide that is conjugated to the glycan strand of peptidoglycan [57], newly remodeled and/or synthesized peptidoglycan can be metabolically labeled using unnatural D-alanine derivatives conjugated to bioorthogonal functional groups [58,59]. To determine if peptidoglycan remodeling and/or synthesis is active during *C*. *difficile* forespore engulfment, we incubated sporulating *C*. *difficile* cultures with D-alanine bearing an alkyne group and visualized its incorporation into peptidoglycan over time using copper-catalyzed click chemistry [59]. Alkyne D-alanine, referred to as “alkDala,” was labeled through the azide-alkyne cycloaddition of an azide group conjugated to a fluorescein-derivative [60].

**Fig 8:**
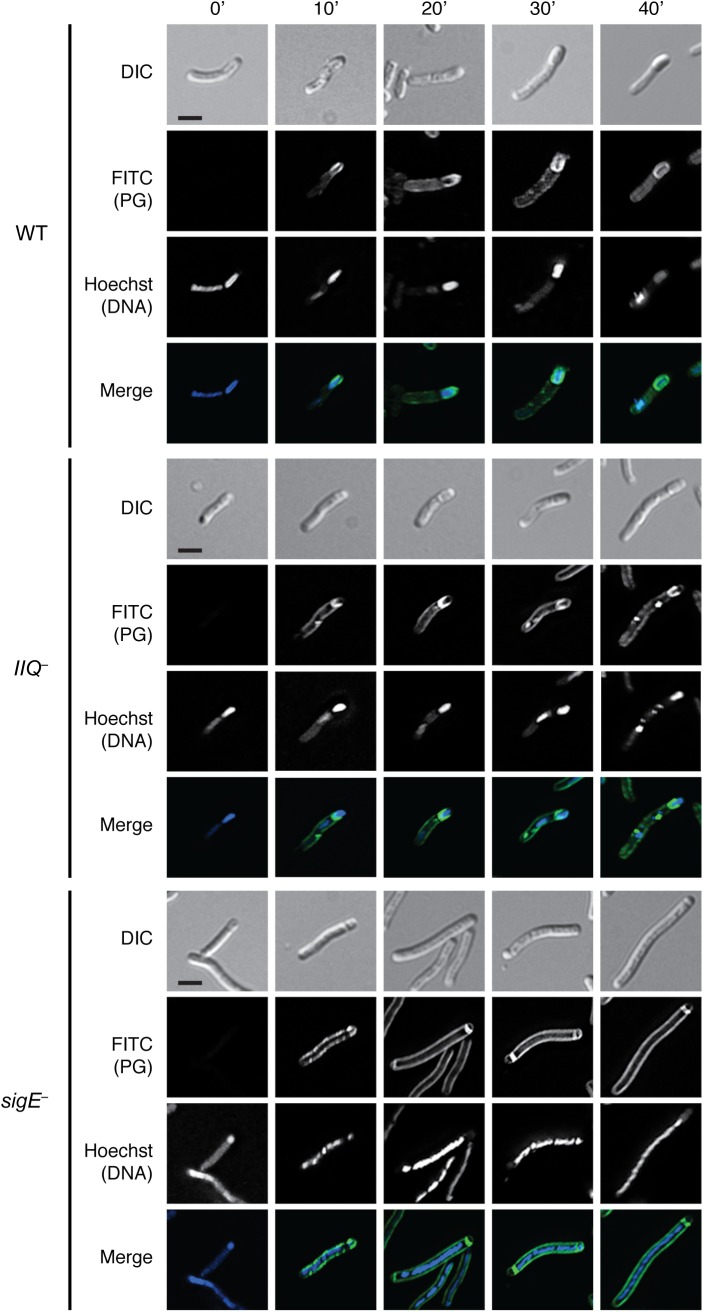
Metabolic labeling of peptidoglycan transformations in WT, *spoIIQ* ^*–*^, and *sigE* ^*−*^strains during sporulation. Strains were induced to sporulate on solid media for 14 hrs then resuspended in liquid sporulation media. Alkyne D-alanine (alkDala) was incubated with the cells for 0’, 10’, 20’, 30’, or 40’. After fixation, permeabilization, and copper-catalyzed cycloaddition of an azide-conjugated fluorophore, Hoechst nucleoid dye was added, and cells were visualized by light microscopy. Scale bars represent 2 μm.

Analysis of wildtype sporulating cells using this metabolic labeling assay revealed that fluorescent peptidoglycan signal (PG) was detectable within 10 min of incubating the culture with alkDala (Figs 8 and S11). After 30 min of incubation with alkDala, the peptidoglycan signal was observed surrounding the forespore and, to a lesser extent, the mother cell (Fig 8). In contrast, when the *spoIIQ* mutant was incubated with alkDala for 10 min or longer, peptidoglycan remodeling and/or synthesis was localized primarily at the curved septa at the mother cell-forespore interface (Fig 8), consistent with the *spoIIQ* mutant’s engulfment defect (Fig 1). Incubation of the *sigE* mutant with alkDala resulted in labeling of the polar septa and mother cell peptidoglycan after 20 min of incubation with alkDala. For comparison, *B*. *subtilis* engulfment requires ~45 min to complete [61,62], and sporulation occurs more slowly in *C*. *difficile* than in *B*. *subtilis* [27,28].

To determine the optimal length of time for measuring alkDala incorporation during sporulation, we analyzed the distribution of the alkDala label in sporulating wild type cells 10’, 20’, 30’, and 40’ after alkDala addition. Sporulating wildtype cells with visible peptidoglycan labeling were binned into the following categories: (i) no staining of the forespore, (ii) labeling of the polar septum, (iii) partial labeling of the forespore on the mother cell distal side (i.e. labeling after engulfment has initiated), (iv) partial labeling around the middle of the forespore, (v) partial labeling of the forespore on the mother cell proximal side, (vi) labeling around the entire forespore (S11 Fig). Based on these analyses, we chose to label cells after a 30 minute incubation with alkDala, since this was the earliest time point at which full labeling of the forespore was detected in the majority of wildtype sporulating cells (S11 Fig).

**Fig 9:**
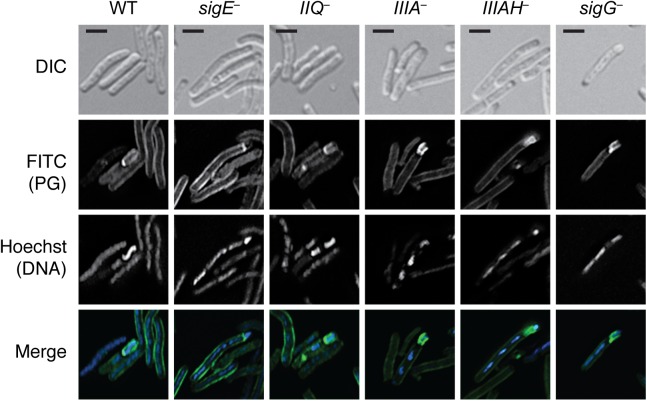
Engulfment defective mutants actively remodel peptidoglycan around the forespore. Strains were induced to sporulate on solid media for 14 hrs then resuspended in liquid sporulation media and incubated with alkyne D-alanine (alkDala). After fixation, permeabilization, and copper-catalyzed cycloaddition of an azide-conjugated fluorophore, Hoechst nucleoid dye was added, and cells were visualized by light microscopy. Scale bars represent 2 μm.

As expected, the alkDala probe labeled division septa in all strains (S12A Fig), and fluorescent labeling was not observed in wildtype cells incubated with D-alanine, which cannot undergo cycloaddition (S12B Fig), or at time 0 min, even though the samples were exposed to the azido-fluorophore probe (Fig 9). To ensure that alkDala specifically labeled newly transformed PG, we incubated WT cells with the cell wall synthesis inhibitors vancomycin and imipenem prior to addition of alkDala and evaluated alkDala incorporation by flow cytometry. Vancomycin inhibits cell wall synthesis by preventing both transpeptidation and transglycosylation [63], and imipenem covalently inhibits the penicillin binding proteins required for transpeptidation [64]. Incubation of wildtype cells with alkDala resulted in a statistically significant increase in median fluorescent intensity (MFI) relative to the MFI of WT cells incubated with Dala (S13 Fig; p < 0.0001). While vancomycin treatment did not reduce alkDala labeling relative to the positive control in a statistically significant manner, imipenem treatment decreased alkDala label incorporation ~5-fold relative to the positive control (p < 0.0001). Taken together, these results suggest that the alkDala probe specifically labels newly synthesized and/or remodeled peptidoglycan, and peptidoglycan continuously surrounds the *C*. *difficile* forespore throughout engulfment as previously observed in *B*. *subtilis* [65].

Since the metabolic labeling time course demonstrated that the fluorescent signal was maximal after 30 min of alkDala incorporation, we used this labeling period to assess whether *C*. *difficile* mutants defective in engulfment could remodel and/or synthesize peptidoglycan around the forespore. Although the metabolic label was evenly distributed around the entire perimeter of wildtype forespores, the label was only partially distributed around the mother cell proximal side of the forespore in engulfment-defective *spoIIQ*, *spoIIIA*, *spoIIIAH*, and *sigG* mutants (Fig 9). These results suggest that the engulfment defect of the *spoIIQ*, *spoIIIA*, *spoIIIAH*, and *sigG* mutants is not due to a failure to activate peptidoglycan remodeling and/or synthesis. Instead, the active peptidoglycan transformations observed in the *spoIIQ*, *spoIIIA*, and *spoIIIAH* mutants appear to be insufficient to drive engulfment to completion.

## Discussion

Since the SpoIIIAA-AH components of the *B*. *subtilis* “feeding tube” channel are universally conserved in spore-forming organisms [31,32], and a SpoIIQ-like ortholog is conserved in the Clostridia [32], we hypothesized that these proteins would play a critical role in regulating *C*. *difficile* spore formation. In this study, we have demonstrated that *C*. *difficile* SpoIIQ and SpoIIIA proteins control forespore engulfment and integrity and the intimate association of the coat with the forespore (Figs 3, 4 and S7). Although SpoIIQ and SpoIIIAH are strongly required for engulfment, the SpoIIIAA-AF proteins appear to be only partially required for engulfment completion in *C*. *difficile*, since the *spoIIIA* mutant completes engulfment ~10–20% of the time (Figs 1, 7, and S7). Given that this mutant produces wildtype levels of SpoIIQ and SpoIIIAH (Fig 2), and SpoIIQ and SpoIIIAH directly interact at least *in vitro* (Fig 6) similar to their *B*. *subtilis* counterparts [34], *C*. *difficile* SpoIIQ and SpoIIIAH would appear to be sufficient to complete engulfment in some *spoIIIA* mutant cells.

While it remains possible that SpoIIIAG also regulates *C*. *difficile* forespore engulfment in the *spoIIIA* mutant, our observations are nevertheless consistent with the proposal that SpoIIQ-SpoIIIAH complex functions like a “Brownian” ratchet to allow for “zipper-like” engulfment [41]. Indeed, the finding that the SpoIIQ H120A mutant exhibits only a partial defect in engulfment completion and heat-resistant spore formation relative to wild type (~50%, S9 Fig) implies that SpoIIQ-SpoIIIAH complex formation is more important for engulfment completion than the putative endopeptidase activity of *C*. *difficile* SpoIIQ. In contrast, *B*. *subtilis* SpoIIQ lacks endopeptidase activity, and the SpoIIQ-SpoIIIAH complex is dispensable for engulfment when sporulation is induced by the re-suspension method [20,41,66]. However, when sporulation is induced by nutrient exhaustion, *B*. *subtilis* SpoIIQ is required to complete engulfment [22,33,41]. This observation suggests that media composition causes changes within sporulating cells such that some sporulation proteins are differentially required for engulfment. Since the 70:30 media used to induce *C*. *difficile* sporulation in this study resembles the nutrient exhaustion media used in *B*. *subtilis* [67], it will be interesting to test whether differences in media compositions and sporulation conditions (e.g. broth vs. plate-based induction) will lead to differential requirements for *C*. *difficile* SpoIIQ and SpoIIIAH during engulfment. Indeed, *C*. *difficile sigG* mutants appear to exhibit differences in engulfment completion when sporulation is induced in broth vs. on plates, although slight differences in strain background could be responsible for this difference [27,28]. Regardless, the engulfment defects of *C*. *difficile spoIIQ* and *spoIIIAH* mutants suggest that the ancestral function of the SpoIIQ-SpoIIIAH complex is to control engulfment during sporulation [68].

Even though SpoIIQ and SpoIIIAH appear to be sufficient to mediate engulfment in ~15% of *C*. *difficile spoIIIA* ^*−*^cells, the *spoIIIA* ^*−*^mutant nevertheless failed to produce heat-resistant spores. Since the *spoIIIA* ^*−*^mutant is likely defective in producing the SpoIIIAA-AF proteins (S4 Fig), these proteins would appear to regulate steps beyond engulfment during *C*. *difficile* sporulation (Fig 1). Indeed, our mutational analyses implicate SpoIIIAA’s predicted ability to bind ATP as being critical for engulfment completion, since mutation of the Walker A ATP binding motif (K167A) results in an ~300-fold defect in heat-resistant spore formation relative to wild type (Fig 7). Interestingly, ATP hydrolysis would appear to be less important for SpoIIIAA’s function during spore formation, since the Walker B mutant (D244A) exhibits only a 3-fold defect in engulfment and heat-resistant spore formation (Fig 7). Given that the phenotype of the K167A/D244A double mutant resembles that of the K167A single mutant, nucleotide binding by SpoIIIAA may induce a conformational change within the protein that is necessary for its function. Consistent with this hypothesis, *B*. *subtilis* SpoIVA Walker A ATP binding mutants exhibit different phenotypes from Walker B ATPase mutants [52]. Alternatively, a different aspartate residue may substitute for the predicted role of D244 in catalyzing *C*. *difficile* SpoIIIAA’s ATPase activity. While this functional redundancy is formally possible, we note that the equivalent Walker B mutation in *B*. *subtilis* SpoIIIAA (D224A) causes a heat-resistant spore formation defect equivalent to a Δ*spoIIIAA* mutant [20]. It will be important in future studies to determine whether *C*. *difficile* SpoIIIAA binds and hydrolyzes ATP, and whether these activities are necessary to power transport of proteins and/or metabolites from the mother cell to the forespore similar to *B*. *subtilis* SpoIIIAA [19–21].

While the *C*. *difficile spoIIIA* mutant completed engulfment in ~10–20% of cells yet failed to produce heat-resistant spores, the *C*. *difficile spoIIQ* mutant exhibited a severe engulfment defect and produced heat-resistant spores 0.5% of the time relative to wild type. The mechanism by which *spoIIQ* ^*−*^cells form functional spores remains mysterious given that SpoIIIAH likely binds SpoIIQ during *C*. *difficile* sporulation (Fig 6) and is required for heat-resistant spore formation. Interestingly, a differential requirement for SpoIIIAH is observed in *B*. *subtilis*, since a *spoIIIAH* mutant has a 1000-fold less severe phenotype relative to other *spoIIIA* mutants [20]. While a mechanism underlying these differential phenotypes remains unclear for both *C*. *difficile* and *B*. *subtilis*, functionally redundant mechanisms appear to exist in both organisms. Testing this hypothesis in *C*. *difficile* would be greatly aided by analyses of *C*. *difficile* SpoIIQ and SpoIIIA protein complex formation during sporulation.

Although the SpoIIQ and SpoIIIA proteins regulate *C*. *difficile* forespore engulfment, these proteins appear dispensable for σ^G^ activity in the forespore (Figs 5 and S8) as predicted [27,29]. Despite these observations, it nevertheless remains possible that these proteins are needed to sustain σ^G^ activity after engulfment is complete. Contrary to this model, the number of *spoIIIA* ^*−*^cells that activated σ^G^ was identical to wild type (Fig 5). Nevertheless, since we cannot assess the duration of σ^G^ activity in the forespore due to the inability to synchronize sporulation in *C*. *difficile* [27,28,45], it remains possible that the forespore may require resources from the mother cell in a SpoIIQ- and SpoIIIA-dependent manner during late stages of sporulation.

Our analyses also uncovered a surprising role for *C*. *difficile* SpoIIQ and SpoIIIA proteins in regulating the adherence of the spore coat to the engulfing forespore. TEM analyses revealed that the spore coat appears to localize and anchor to the leading edge of the engulfing membrane but sometimes sloughs away from the mother cell-forespore interface (Fig 3). Unfortunately, little is known about the mechanisms by which the spore coat localizes around the forespore in *C*. *difficile*, since few coat morphogenetic proteins are conserved between *C*. *difficile* and *B*. *subtilis* [18]. SpoIVA and the clostridial-specific SipL have been shown to function as coat morphogenetic proteins by localizing the coat to the forespore in *C*. *difficile* [45], but how these proteins are recruited to the forespore membrane is unclear. Our results suggest an intriguing link between engulfment completion and adhering coat around the forespore, since the minority of *spoIIIA* ^*−*^and *spoIIQ* ^*−*^cells that completed engulfment produced coat surrounding the forespore (S7 Fig). Perhaps proteins localized to the leading edge of the engulfing membrane recruit *C*. *difficile* coat proteins but are insufficient to adhere the coat to the forespore in the absence of engulfment completion, or mechanical forces that drive engulfment to completion are also required to intimately associate the coat with the forespore.

A link between SpoIIQ and coat localization around the forespore has been described in *B*. *subtilis*, since SpoIIQ is required for many coat proteins, including the σ^E^-dependent coat protein CotE (unrelated to *C*. *difficile* CotE [47]), to surround the forespore in a process known as “encasement” [69]. Since *B*. *subtilis* CotE localizes properly in a *sigG* mutant [69], the *B*. *subtilis spoIIQ* mutant’s ~30-fold encasement defect suggests that components of the coat indirectly interact with the forespore-localized SpoIIQ. It should be noted, however, that CotE in a *B*. *subtilis spoIIQ* mutant appears to track along the mother cell-forespore interface [69], in contrast with *C*. *difficile spoIIQ*, *spoIIIA*, *and spoIIIAH* mutants in which coat is located some distance from this interface due to an apparent defect in adhering to the forespore (Figs 3 and 4). Furthermore, the *B*. *subtilis* Δ*spoIIQ* mutant completes engulfment in the conditions used for the coat localization studies, whereas the *C*. *difficile spoIIQ* mutant largely fails to complete engulfment (Figs 3, S7 and S9). Since *B*. *subtilis* CotE localization around the forespore depends upon earlier morphogenetic proteins SpoVM, SpoIVA, and SpoVID [69], it would be interesting to determine whether *B*. *subtilis “*feeding tube” components affect the localization of these earlier morphogenetic proteins, and vice versa. Similarly, *C*. *difficile* CotE is a σ^K^-regulated protein that appears to localize to the outermost layers of *C*. *difficile* spores [46], so determining the localization patterns of SpoIVA and/or SipL in the *spoIIQ*, *spoIIIA*, and *spoIIIAH* mutants may provide insight into whether SpoIIQ and/or SpoIIIAA-AH regulate the localization of these coat morphogenetic proteins. Future studies evaluating whether these proteins form a channel in *C*. *difficile*, why these proteins are important for forespore integrity, and how these proteins regulate engulfment and coat association with the forespore will provide much-needed insight into how these cellular processes are controlled in *C*. *difficile* and potentially other spore-forming organisms.

## Materials and Methods

### Bacterial strains and growth conditions

All *C*. *difficile* strains are listed in Table 1 and derive from the parent strain JIR8094, an erythromycin-sensitive derivative [70] of the sequenced clinical isolate 630 [71]. *C*. *difficile* strains were grown on solid brain heart infusion media supplemented with yeast extract (BHIS: 37 g brain heart infusion, 5 g yeast extract, 0.1% (w/v) *L*-cysteine, 15 g agar per liter) [72]. Taurocholate (TA; 0.1% w/v), thiamphenicol (5–10 μg/mL), kanamycin (50 μg/mL), cefoxitin (16 μg/mL), FeSO_4_ (50 μM), and/or erythromycin (10 μg/mL) were used to supplement the BHIS media as indicated. Cultures were grown at 37°C, under anaerobic conditions using a gas mixture containing 85% N_2_, 5% CO_2_, and 10% H_2_.

**Table 1:** *C*. *difficile* strains used in this study.

Strain	*C*. *difficile* strain	Relevant genotype or features
11	JIR8094	erm-sensitive derivative of 630 [71]
13	630	Clinical isolate 630 [72]
35	*spo0A* ^*–*^	JIR8094 *spo0A*::*ermB*
50	*sigE* ^*–*^	JIR8094 *sigE*::*ermB*
67	*sigK* ^*–*^	JIR8094 *sigK*::*ermB*
71	JIR8094/pMTL84151	JIR8094/pMTL84151
99	*sigG* ^–^	JIR8094 *sigG*::*ermB*
106	*sigF* ^*–*^	JIR8094 *sigF*::*ermB*
111	JIR8094/pMTL83151	JIR8094/pMTL83151
218	JIR8094 *spo0A* ^*–*^/pMTL83151	JIR8094 *spo0A*::*ermB*/pMTL83151
251	*spoIIIA* ^*–*^	JIR8094 *spoIIIAA*::*ermB*
253	*spoIIQ* ^–^	JIR8094 *spoIIQ*::*ermB*
295	*spoIIQ* ^–^/pMTL83151-*spoIIQ*	JIR8094 *spoIIQ*::*ermB*/pMTL83151-*spoIIQ*
297	*spoIIIA* ^–^/pMTL83151-*spoIIIA*	JIR8094 *spoIIIAA*::*ermB*/pMTL83151-*spoIIIA* operon
307	*spoIIIA* ^–^/pMTL83151	JIR8094 *spoIIIAH*::*ermB*/pMTL83151
331	*spoIIQ* ^–^/pMTL83151	JIR8094 *spoIIQ*::*ermB*/pMTL83151
415	*spoIIIAH* ^–^	JIR8094 *spoIIIAH*::*ermB*
433	*spoIIIAH* ^*–*^ */pMTL83151-spoIIIA*	JIR8094 *spoIIIAH*::*ermB*/pMTL83151-*spoIIIA* operon
441	*spoIIIA* ^*–*^ */pMTL83151-spoIIIA* K167A	JIR8094 *spoIIIAA*::*ermB*/pMTL83151-*spoIIIA* operon K167A
449	*spoIIIAH* ^–^/pMTL83151	JIR8094 *spoIIIAH*::*ermB*
525	*spoIIIAH* ^*–*^ */*pMTL83151-*spoIIIAH*	JIR8094 *spoIIIAH*::*ermB*/pMTL83151-*spoIIIAH*
540	*sigG* ^–^/pMTL84121-*cotE*-*SNAP*	JIR8094 *sigG*::*ermB*/pMTL84121-*cotE*-*SNAP*
542	*spoIIQ* ^*–*^ */*pMTL84121-*cotE-SNAP*	JIR8094 *spoIIQ*::*ermB*/pMTL84121-*cotE-SNAP*
546	*spoIIIA* ^*–*^ */*pMTL84121-*cotE-SNAP*	JIR8094 *spoIIIAA*::*ermB*/pMTL84121-*cotE-SNAP*
570	JIR8094/pMTL84121-*cotE*-*SNAP*	JIR8094/pMTL84121-*cotE*-*SNAP*
591	*spoIVA* ^–^/pMTL84121-*cotE*-*SNAP*	JIR8094 *spoIVA*::*ermB*/pMTL84121-*cotE*-*SNAP*
608	JIR8094/pMTL84151-P_*sspA*_-*SNAP*	JIR8094/pMTL84151-P_*sspA*_-*SNAP*
611	*sigE* ^–^/pMTL84151-P_*sspA*_-*SNAP*	JIR8094 *sigE*::*ermB*/pMTL84151-P_*sspA*_-*SNAP*
614	*sigG* ^–^/pMTL84151-P_*sspA*_-*SNAP*	JIR8094 *sigG*::*ermB*/pMTL84151-P_*sspA*_-*SNAP*
617	*spoIIIA* ^–^/pMTL84151-P_*sspA*_-*SNAP*	JIR8094 *spoIIIAA*::*ermB*/pMTL84151-P_*sspA*_-*SNAP*
633	*spoIIIAH* ^–^/pMTL84151-P_*sspA*_-*SNAP*	JIR8094 *spoIIIAH*::*ermB*/pMTL84151-P_*sspA*_-*SNAP*
634	*spoIIQ* ^–^/pMTL84151-P_*sspA*_-*SNAP*	JIR8094 *spoIIQ*::*ermB*/pMTL84151-P_*sspA*_-*SNAP*
636	*sigF* ^–^/pMTL84151-P_*sspA*_-*SNAP*	JIR8094 *sigF*::*ermB*/pMTL84151-P_*sspA*_-*SNAP*
649	*spoIIIAH* ^–^/pMTL84121-*cotE*-*SNAP*	JIR8094 *spoIIIAH*::*ermB*/pMTL84121-*cotE*-*SNAP*
752	*spoIIQ* ^*–*^ */*pMTL83151-*spoIIQ* H120A	JIR8094 *spoIIQ*::*ermB*/pMTL83151-*spoIIQ* H120A
760	*spoIIIA* ^*–*^ */*pMTL83151-*spoIIIA* D244A	JIR8094 *spoIIIAA*::*ermB*/pMTL83151-*spoIIIA* operon D244A
763	*spoIIIA* ^*–*^ */*pMTL83151-*spoIIIA* K167A/D244A	JIR8094 *spoIIIAA*::*ermB*/pMTL83151-*spoIIIA* operon K167A/D244A

Sporulation was induced on media containing BHIS and SMC (90 g BactoPeptone, 5 g protease peptone, 1 g NH_4_SO_4_, 1.5 g Tris base, 15 g agar per liter) [73], at 70% SMC and 30% BHIS (70:30 media, 63 g BactoPeptone, 3.5 g Protease Peptone, 11.1 g BHI, 1.5 g yeast extract, 1.06 g Tris base, 0.7 g NH_4_SO_4_, 15 g agar per liter) [45]. 70:30 agar (supplemented as appropriate with thiamphenicol at 10 μg/mL) was inoculated from a starter culture grown on solid media. 70:30 broth was made as stated above omitting the agar.

HB101/pK424 strains were used for conjugations and BL21(DE3) strains were used for protein expression. *E*. *coli* strains were routinely grown at 37°C, shaking at 225 rpm in Luria-Bertani broth (LB). Media was supplemented with chloramphenicol (20 μg/mL), ampicillin (100 μg/mL), or kanamycin (30 μg/mL) as indicated.

### *E*. *coli* strain construction

All strains are listed in S1 Table; all plasmids are listed in S2 Table; and all primers used are listed in S3 Table. For disruption of *spoIIQ*, *spoIIIAA*, *and spoIIIAH*, a modified plasmid containing the retargeting group II intron, pCE245 (a gift from C. Ellermeier, University of Iowa), was used as the template. Primers used to amplify the targeting sequence from the template carried flanking regions specific for each gene target and are listed as follows: *spoIIQ* (#1052, 1053, 1054 and 532, the EBS Universal primer as specified by the manufacturer (Sigma Aldrich)), *spoIIIAA* (#1049, 1050, 1051 and 532), and *spoIIIAH* (#1264, 1265, 1266, and 532). The resulting retargeting sequences were digested with BsrGI and HindIII and cloned into pJS107 (a gift from J. Sorg, University of Texas A&M), a derivative of pJIR750ai (Sigma Aldrich). The ligations were transformed into DH5α and confirmed by sequencing. The resulting plasmids were used to transform HB101/pK424.

To construct the *spoIIQ* complementation construct, primers #1177 and 1178 were used to amplify *spoIIQ* containing 106 bp of the upstream region using 630 genomic DNA as the template. To construct the *spoIIQ* H120A complementation construct, SOE primers #1177 and #1851 were used to generate a 5’ fragment (590 bp) containing the H120A mutation; primers #1850 and #1178 were used for the 3’ SOE product using the *IIQ* complementation construct as a template. To construct the *spoIIIA* operon complementation construct, primers #1174 and 1175 were used to amplify 211 bp upstream of *spoIIIAA* and 9 bp downstream of *spoIIIAH* using 630 genomic DNA as the template. The *spoIIIAH* complementation construct was made using PCR splicing by overlap extension (SOE, [74]). Primer pair #1174 and 1618 was used to amplify the 5’ SOE product, while primer pair #1617 and 1239 was used to amplify the 3’ SOE product. The resulting fragments were mixed together, and flanking primers #1174 and #1239 were used to generate a fragment corresponding to 211 bp of the *spoIIIA* upstream region fused to the *spoIIIAH* gene (P_*spoIIIA*_-*spoIIIAH*). To construct the *spoIIIA* operon K167A complementation construct, SOE primers #1174 and #1432 were used to generate a 5’ fragment (590 bp) containing the K167A mutation; primers #1431 and #1175 were used for the 3’ SOE product. The flanking primers #1174 and #1175 were used to amplify the K167A *IIIA* complementation construct. To construct the *spoIIIA* operon D244A complementation construct, SOE primers #1174 and #1853 were used to generate a 5’ fragment (590 bp) containing the D244A mutation; primers #1852 and #1854 were used for the 3’ SOE product using the *IIIA* complementation construct as a template. The flanking primers #1174 and #1854 were used to amplify the D244A *IIIA* mutation insert, digested with NotI and SalI. The plasmid carrying the *IIIA* complementation construct was also digested with NotI/SalI and then gel purified to separate the plasmid backbone from the wildtype fragment. The D244A *IIIA* NotI/SalI fragment was ligated to the gel-purified cut vector. To construct the *spoIIIA* operon K167A/D244A complementation construct, SOE primers #1174 and #1853 were used to generate a 5’ fragment (590 bp) containing the D244A mutation; primers #1852 and #1854 were used for the 3’ SOE product using the K167A complementation construct as a template. The flanking primers #1174 and #1854 were used to amplify the D244A *IIIA* mutation insert, digested with NotI and SalI, and ligated to the *IIIA* complementation construct digested with NotI/SalI as described earlier. All complementation constructs except for the D244A and K167A/D244A were digested with NotI and XhoI and ligated into pMTL83151 [44] digested with the same enzymes.

To construct strains producing recombinant N-terminally truncated SpoIIQ and N-terminally truncated SpoIIIAH for antibody production, primer pairs #1568 and 1569 and #1566 and 1567, respectively were used to amplify codon optimized *spoIIQ* and *spoIIIAH* genes lacking stop codons off template synthesized by Genscript. The *spoIIQ* expression construct deletes the sequence encoding the first 30 amino acids of SpoIIQ, while the *spoIIIAH* expression construct deletes the sequence encoding the first 33 amino acids of SpoIIIAH, which removes the membrane-tethering domains and improves the solubility of the proteins in *E*. *coli*. The resulting PCR products were digested with NdeI and XhoI, ligated to pET22b, and transformed into DH5α. The resulting pET22b-*spoIIQ* and pET22b-*spoIIIAH* plasmids were used to transform BL21(DE3) for protein expression.

To construct the pET28a-HA-*spoIIIAH* construct for the affinity co-purification studies, primer pair #1665 and 1614 was used on the codon-optimized *spoIIIAH* template synthesized by Genscript. To construct the pET28a-HA-*spoVT* construct for the affinity co-purification studies, primer pair #1691 and 1313 was used to amplify *spoVT* encoding an N-terminal HA-tag using *C*. *difficile* genomic DNA as the template. The resulting PCR products were digested with NcoI and XhoI, ligated to pET28a digested with the same enzymes, and transformed into DH5α. The pET28a-HA-*spoIIIAH* construct was transformed into BL21(DE3) to construct strain #1378. The pET28a-HA-*spoIIIAH* construct was transformed into BL21(DE3) to construct strain #1404.

To construct the σ^G^-dependent transcriptional reporter, the σ^G^-regulated promoter of *sspA* (P_*sspA*_) was fused to a *C*. *difficile* codon optimized SNAP-tag [75] to generate P_*sspA*_-SNAP (Genscript) with flanking restriction sites. This promoter region has previously been described [28]. The plasmid was transformed into *E*. *coli* DH5α, isolated, and digested with NotI and XhoI then cloned into the complementation plasmid pMTL84151, transformed into *E*. *coli* HB101 (S1 Table) and subsequently conjugated into *C*. *difficile* strains.

### *C*. *difficile* strain construction

*C*. *difficile* strains were constructed using TargeTron-based gene disruption as described previously (S4 Fig, [27]). TargeTron constructs in pJS107 were conjugated into *C*. *difficile* using an *E*. *coli* HB101/pK424 donor strain. HB101/pK424 strains containing the appropriate pJS107 construct were grown aerobically to exponential phase in 2 mL of LB supplemented with ampicillin (50 μg/mL) and chloramphenicol (10 μg/mL). Cultures were pelleted, transferred into the anaerobic chamber, and resuspended in 1.5 mL of late-exponential phase *C*. *difficile* JIR8094 cultures (grown anaerobically in BHIS broth). The resulting cell mixture was plated as seven 100 μL spots onto pre-dried, pre-reduced BHIS agar plates. After overnight incubation, all growth was harvested from the BHIS plates, resuspended in 2.5 mL pre-reduced BHIS, and twenty-one 100 μL spots per strain were plated onto three BHIS agar plates supplemented with thiamphenicol (10 μg/mL), kanamycin (50 μg/mL), and cefoxitin (16 μg/mL) to select for *C*. *difficile* containing the pJS407 plasmid. After 24–48 hrs of anaerobic growth, single colonies were patched onto BHIS agar supplemented with thiamphenicol (10 μg/mL), kanamycin (50 μg/mL), and FeSO_5_ (50 μM) to induce the ferredoxin promoter of the group II intron system. After overnight growth, patches were transferred to BHIS agar plates supplemented with erythromycin (10 μg/mL) for 24–72 hrs to select for cells with activated group II intron systems. Erythromycin-resistant patches were struck out for isolation onto the same media and individual colonies were screened by colony PCR for a 2 kb increase in the size of *spoIIQ* (primer pair #1074 and 1075), *spoIIIAA* (primer pair #1302 and 1176), and *spoIIIAH* (primer pair #1301 and 1239) (S4 Fig).

### *C*. *difficile* complementation

HB101/pK424 donor strains carrying the appropriate complementation construct were grown in LB containing ampicillin (50 μg/mL) and chloramphenicol (20 μg/mL) at 37°C, 225 rpm, under aerobic conditions, for 6 hrs. *C*. *difficile* recipient strains *spoIIQ* ^*–*^, *spoIIIAA* ^*–*^, and *spoIIIAH* ^*−*^containing group II intron disruptions, were grown anaerobically in BHIS broth at 37°C with gentle shaking for 6 hrs. HB101/pK424 cultures were pelleted at 2500 rpm for 5 min and the supernatant was removed. Pellets were transferred to the anaerobic chamber and gently resuspended in 1.5 mL of the appropriate *C*. *difficile* culture. The resulting mixture was inoculated onto pre-dried, pre-reduced BHIS agar plates, as seven 100 μL spots for 12 hrs. All spots were collected anaerobically and resuspended in 1 mL PBS. The resulting suspension was spread onto pre-dried, pre-reduced BHIS agar plates supplemented with thiamphenicol (10 μg/mL), kanamycin (50 μg/mL), and cefoxitin (10 μg/mL) at 100 μL per plate, five plates per conjugation. Plates were monitored for colony growth for 24–72 hrs. Individual colonies were struck out for isolation and analyzed for complementation by phase contrast microscopy, Western blot analysis and transmission electron microscopy. A minimum of two independent clones from each complementation strain was phenotypically characterized.

For the SNAP-tag expression constructs, a pMTL84151 [44] or pMTL84121 [28] plasmid backbone was used. The complementation protocol was followed as described except that after spots were collected from overnight growth on BHIS plates, 100 μL of the resulting PBS suspension was spotted 7 times onto a BHIS agar plate supplemented with thiamphenicol (10 μg/mL), kanamycin (50 μg/mL), and cefoxitin (16 μg/mL). This procedure was repeated for three plates.

### Sporulation assay

*C*. *difficile* strains were grown from glycerol stocks on BHIS plates supplemented with TA (0.1% w/v), or with both TA and thiamphenicol (5–10 μg/mL) for strains with pMTL83151-derived or pMTL84151-derived plasmids (as previously described [27]). Cultures grown on BHIS agar plates were then used to inoculate 70:30 agar plates (with thiamphenicol at 5–10 μg/mL as appropriate) for 17–24 hrs depending on the assay. Sporulation induced lawns were harvested in PBS, washed once, resuspended in PBS, visualized by phase contrast microscopy, and/or further processed for analysis by transmission electron microscopy, Western blotting, or fluorescence microscopy.

### Heat resistance assay

*C*. *difficile* strains were induced to sporulate as described above, and cells were harvested in 1.0 mL PBS, and split into two tubes. One tube was heat shocked at 60–65°C for 25 minutes. Both heat-shocked and non-heat shocked cells were serially diluted, and cells were plated on pre-reduced BHIS-TA plates. After 20 hrs on BHIS-TA, colonies were counted, and cell counts were determined. The percent of heat-resistant spores was determined based on the ratio of heat-resistant cells to total cells, and sporulation efficiencies were determined based on the ratio of heat-resistant cells for a strain compared to wild type. Results are based on a minimum of three biological replicates.

### Electron microscopy

One hundred microliters of bacterial cell suspension samples from sporulation assays were prepared as previously described [45].

### Antibody production

The anti-SpoIIQ and anti-SpoIIIAH antibodies used in this study were raised in rabbits by Cocalico Biologicals (Reamstown, PA). The antigens SpoIIQ-His_6_ and SpoIIIAH-His_6_ were purified on Ni^2+^-affinity resin from *E*. *coli* strains #1301 and 1302 as described above. Cultures were grown and protein expression was analyzed as previously described [27].

### Western blot analyses

Sporulation assay *C*. *difficile* cells (50 μL of PBS suspension) were freeze-thawed three times, diluted in 100 μL EBB buffer (8 M urea, 2 M thiourea, 4% (w/v) SDS, 2% (v/v) β-mercaptoethanol), and incubated at 95°C for 20 min with vortexing every 5 min. Samples were centrifuged for 5 min at 15,000 rpm, and 7 μL of 4X sample buffer (40% (v/v) glycerol, 1 M Tris pH 6.8, 20% (v/v) β-mercaptoethanol, 8% (w/v) SDS, and 0.04% (w/v) bromophenol blue), was added. Protein samples were incubated again at 95°C for 15 minutes with vortexing followed by centrifugation for 5 min at 15,000 rpm. SDS-PAGE gels (12%–15%) were loaded with 5 μL of the sample. Gels were transferred to Bio-Rad PVDF membrane and blocked in 50% PBS:50% Odyssey Blocking Buffer with 0.1% (v/v) Tween for 30 min at RT. Polyclonal rabbit anti-SpoVT ([27] anti-SpoIIQ and anti-SpoIIIAH, antibodies were used at a 1:1,000 dilution. Monoclonal mouse anti-Spo0A [27] was used at a 1:10,000 dilution. Monoclonal mouse anti-SNAP (NEB) was used at a 1:2,000 dilution. IRDye 680CW and 800CW infrared dye-conjugated secondary antibodies were used at a 1:20,000 dilutions. The Odyssey LiCor CLx was used to detect secondary antibody fluorescent emissions for Western blots.

### RNA processing

RNA from WT, *spo0A* ^*–*^, *sigF* ^*–*^, *spoIIQ* ^*–*^, *sigE* ^*–*^, *spoIIIA* ^*–*^, *spoIIIAH* ^*–*^, and *sigG* ^*−*^strains grown for 17 hrs on 70:30 sporulation media was extracted for qRT-PCR analyses of *spoIIIAA* transcript. RNA from WT, *spo0A* ^*–*^, *sigF* ^*–*^, *spoIIQ* ^*–*^, *sigE* ^*–*^, *spoIIIA* ^*–*^, and *sigG* ^*−*^strains grown for 25 hr on 70:30 sporulation media was extracted for qRT-PCR analyses of *spoVT*, *CD1430*, and *spoVAD* transcripts. RNA from WT/EV, *spo0A* ^*–*^ */*EV, *spoIIIAA* ^*–*^ */*EV, *spoIIIA* ^*–*^ */spoIIIA* operon, and *spoIIIA* ^*–*^ */spoIIIA*K167A operon complementation strains grown for 17 hr on 70:30 sporulation media was extracted for qRT-PCR analyses of *spoIIIAA* transcript. RNA was extracted using a FastRNA Pro Blue Kit (MP Biomedical) and a FastPrep-24 automated homogenizer (MP Biomedical). Contaminating genomic DNA was depleted using three successive DNase treatments and mRNA enrichment was done using an Ambion MICROB*Express* Bacterial mRNA Enrichment Kit (Invitrogen). Samples were tested for genomic DNA contamination using quantitative PCR for *rpoB*. Enriched RNA was reverse transcribed using Super Script First Strand cDNA Synthesis Kit (Invitrogen) with random hexamer primers.

### Quantitative RT-PCR

Transcript levels of *spoIIIAA* and *rpoB* (housekeeping gene) were determined from cDNA templates prepared from 3 biological replicates of WT, *spo0A* ^*–*^, *sigF* ^*–*^, *spoIIQ* ^*–*^, *sigE* ^*–*^, *spoIIIA* ^*–*^, *spoIIIAH* ^*–*^, and *sigG* ^*−*^and three biological replicates of WT/EV, *spo0A* ^*–*^ */*EV, *spoIIIA* ^*–*^ */*EV, *spoIIIA* ^*–*^ */spoIIIA* operon, and *spoIIIA* ^*–*^ */spoIIIA*K167A operon. Gene-specific primer pairs for *spoIIIAA* and *rpoB* have been previously described [29,75]. Transcript levels of *spoVT*, *CD1430*, *spoVAD*, and *rpoB* were determined from cDNA templates prepared from three biological replicates of WT, *spo0A* ^*–*^, *sigE* ^*–*^, *spoIIIA* ^*–*^, and *sigG* ^*–*^. Transcript levels of *CD1430* and *spoVAD* were analyzed using gene-specific primer pairs #1458 and 1459, #1708 and 1709, respectively. Gene-specific primers for measuring *spoVT* transcript levels have been previously described [27]. Quantitative real-time PCR was performed (as described by [75]). Briefly, using SYBR Green JumpStart Taq Ready Mix (Sigma), 50 nM of gene specific primers, and an ABI PRISM 7900HT Sequence Detection System (Applied Biosystems). Transcript levels were normalized to the housekeeping gene *rpoB* using the standard curve method and calculated relative to either the *spo0A* ^*–*^ strain or *spo0A* ^*–*^ strain carrying empty pMTL83151 vector.

### SNAP-tag reporter construction

The CotE-SNAP previously described by Pereira et al. [28] was transformed into *E*. *coli* HB101/ pK424 and conjugated into the indicated *C*. *difficile* strains to analyze coat localization in *spoIIQ* and *spoIIIA* mutants.

*C*. *difficile* strains containing SNAP-tag reporters were grown on 70:30 media to induce sporulation. Cells were grown as a lawn for 21 hours on solid 70:30 media and harvested as described by Pereira *et al*. [28]. Briefly, cells were harvested in PBS and pelleted (4,000 rpm for 3 min), washed once with PBS, reconstituted in 100 μL of PBS. TMR-star SNAP substrate (NEB) was added to a final concentration of 3 μM to each tube and cells were incubated for 30 min at 37°C. Cells were pelleted, washed 3 times with PBS, and resuspended in PBS. Hoechst 33342 (10 mg/ml) was added to a final concentration of 15 μg/ml and FM4-64 (200 μg/ml) was added to a final concentration of 1 μg/mL.

### Peptidoglycan labeling assay

Strains were harvested from 70:30 plates after 14 hours of growth as a bacterial lawn and re-suspended in 3 mL of 70:30 liquid media. For each strain used, the culture was split into 2 tubes for two conditions, each containing 1.5 mL of culture. Alkyne D-alanine or D-alanine (ACROS Organics) was added to each tube, respectively, at a final concentration of 2.5 mM and incubated at 37°C for 30 min. with mild shaking. After incubation, cells were pelleted (8000 rpm for 3 min.) and washed 3x with PBS. Cells were resuspended in 0.7 mL of 2% formaldehyde diluted in PBS and incubated for 10 min on the nutator. Cells were then pelleted and washed 2x with 1 mL PBS. Cells were incubated with 5 mg/mL lysozyme, 37°C, 45 min., pelleted, washed 2x with 1 mL PBS, and washed once with 3% BSA (in PBS). For the click chemistry reaction, a Click-iT Plus Alexa Fluor 488 Picolyl Azide Toolkit (Molecular Probes) was used according to the manufacturer’s instructions. After incubation with Click-iT reagents, samples were pelleted and washed 1x with 3% BSA and 1x with PBS. Samples were resuspended and Hoechst 33342 (10 mg/ml) was added to a final concentration of 15 μg/ml.

For the peptidoglycan labeling timecourse, cells were harvested into 5.5 ml of 70:30 broth after 14 hours of growth on 70:30 plates, and either alkyne D-alanine or D-alanine (ACROS Organics) was added to each tube. One mL samples of each culture was taken at every timepoint (0, 10, 20, 30, and 40 min) and processed as described above.

For evaluation of peptidoglycan labeling after treatment with antibiotics, 1 ml of WT cells in BHIS broth were harvested at late exponential phase for each treatment condition. 2X MIC of antibiotics (2 μg/ml vancomycin and 8 μg/ml imipenem) was added to designated cells and mixed with mild shaking. Dala and alkDala was added immediately after to designated cells and incubated for 30 minutes with mild shaking in the anaerobic chamber. Cells were processed for peptidoglycan labeling as described above.

### Flow cytometry

The median fluorescent intensity (MFI) of alkDala incorporation was determined using a MACSQuant VYB flow cytometer. MACSQuantify software was used for data collection and FlowJo V.10.0.8 was used for data analysis. Cells that incorporated Hoechst dye 33342 (Molecular Probes) were evaluated for alkDala staining based on fluorescence in the FITC channel.

### His_6_-tag pulldowns

*E*. *coli* BL21(DE3) strains were grown to mid-log phase in 2YT (5 g NaCl, 10 g yeast extract, and 15 g tryptone per liter), 225 rpm, at 37°C. 250 μM isopropyl-β-D-1-thiogalactopyranoside (IPTG) was added to induce the cells followed by an overnight incubation at 18°C. Cultures were pelleted, resuspended in low-imidazole buffer (500 mM NaCl, 50 mM Tris [pH 7.5], 15 mM imidazole, 10% [vol/vol] glycerol), and lysed by freeze-thawing and sonication. The insoluble material was pelleted, and the soluble fraction (Input) was batch affinity purified using Ni^2+^ affinity resin and eluted with high-imidazole buffer (500 mM NaCl, 50 mM Tris [pH 7.5], 150 mM imidazole, 10% [vol/vol] glycerol). The resulting eluates were run on SDS-PAGE gels (12%) and transferred onto a PVDF membrane for Western blot analysis, as described above.

### Fluorescence microscopy

For live cell fluorescence microscopy studies, *C*. *difficile* strains were harvested in PBS, pelleted, and resuspended in PBS. For initial characterization of mutant phenotypes, cells were resuspended in PBS containing 1 μg/mL FM4-64 (Molecular Probes) and 15 μg/mL Hoechst 33342 (Molecular Probes). All live bacterial suspensions (4 μL) were added to a freshly prepared 1% agarose pad on a microscope slide, covered with a 22 x 22 mm #1 coverslip and sealed with VALAB (1:1:1 of vaseline, lanolin, and beeswax) as previously described [27].

DIC and fluorescence microscopy was performed using a Nikon PlanApo Vc 100x oil immersion objective (1.4 NA) on a Nikon Eclipse Ti2000 epifluorescence microscope. Multiple fields for each sample were acquired with an EXi Blue Mono camera (QImaging) with a hardware gain setting of 1.0 and driven by NIS-Elements software (Nikon). Images were subsequently imported into Adobe Photoshop CS6 for minimal adjustments in brightness/contrast levels and pseudocoloring.

DIC and fluorescence microscopy for cells that were processed for peptidoglycan labeling experiments were performed and processed with the same equipment as described above with the following differences: a Nikon PlanApo Vc 60x oil immersion objective (1.4 NA) was utilized, hardware gain setting 2.0, and fields were imaged with Z-spacing of 0.15 μm followed by deconvolution using AutoQuant 3x software (MediaCybernetics).

Quantification of total cells undergoing sporulation was determined by analyzing multiple fields for each strain at random. At least 50 cells were enumerated for each strain. Sporulating cells were identified as either having a polar septum with or without DNA staining in the forespore, a DIC-dark forespore with or without DNA staining in the forespore compartment, a DIC-bright forespore without DNA staining, or a free spore (no mother cell compartment).

## Supporting Information

S1 Fig ClustalW sequence alignment of SpoIIIAA. Completely conserved, identical residues are blocked in blue, conserved identical residues are blocked in green, and conserved similar residues in yellow. The conserved motifs (boxes) found in all secretion NTPases are highlighted [20]. The conserved lysine in the Walker A motif that was mutated (K167 in *C*. *difficile* SpoIIIAH) and conserved aspartate in the Walker B motif (D244 in *C*. *difficile* SpoIIIAH) are shown boxed in orange. SpoIIIAA sequences are from *B*. *subtilis* serovar *subtilis* str. 168 (CAA43959) *B*. *cereus* serovar *anthracis* (YP_003793897), *C*. *botulinum* ATCC 3502 (CAL83436), *C*. *acetobutylicum* ATCC 824 (AE007711_9), *C*. *perfringens* str. 13 (NP_562749), *C*. *bartlettii* CAG 1329 (WP_022072529), *C*. *bifermentans* ATCC 19299 (EQK45222), C. *sordellii* VPI 9048 (EPZ57018), and *C*. *difficile* 630 (YP_001087685). *C*. *bartlettii*, *C*. *bifermentans*, *C*. *sordellii* and *C*. *difficile* are part of the *Peptoclostridium* spp. [78].
(TIF)

S2 Fig ClustalW sequence alignment of SpoIIIAH. Completely conserved, identical residues are blocked in blue, conserved identical residues are blocked in green, and conserved similar residues in yellow. α-helices and β-sheets in the *B*. *subtilis* SpoIIIAH (NP_390316) extracellular domain (determined from the SpoIIQ-SpoIIIAH complex, [37,38]) are indicated as a helix or black arrow above the sequence alignment, respectively, with the first and last α-helices and β-sheets being labeled. Purple asterisks identify *B*. *subtilis* SpoIIIAH residues that directly interact with SpoIIQ as determined by both Levdikov *et al*. and Meisner *et al*. [37,38]. Orange asterisks indicate *B*. *subtilis* SpoIIIAH residues that directly interact with SpoIIQ as determined by Levdikov *et al*. [37]. The remaining sequences are from *B*. *cereus* serovar *anthracis* (YP_003793890), *C*. *botulinum* ATCC 3502 (YP_001254390), *C*. *acetobutylicum* ATCC 824 (AE007711_2), *C*. *perfringens* str. 13 (NP_562742), *C*. *bartlettii* CAG 128 (CDA09218), *C*. *bifermentans* ATCC 19299 (EQK45176), C. *sordellii* VPI 9048 (EPZ57011), and *C*. *difficile* 630 (YP_001087692). *C*. *bartlettii*, *C*. *bifermentans*, *C*. *sordellii* and *C*. *difficile* are part of the *Peptoclostridium* genus [78].
(TIF)

S3 Fig ClustalW sequence alignment of SpoIIQ orthologs of CD0125. Orthologs were identified based on a Hidden Markov Model search using HHMER [79]. Completely conserved, identical residues are blocked in blue, conserved identical residues are blocked in green, and conserved similar residues in yellow. A red triangle demarcates active site residues (boxed in red), with phosphate binding residues in the active site being marked with a blue triangle (boxed in blue) [32]. The intact active site His in the HxxxD motif of the metallopeptidase found in *C*. *difficile* CD0125 is shown in red; the active site His has been mutated to Ser in *B*. *subtilis* SpoIIQ. Residue numbering is based on *B*. *subtilis* SpoIIQ (NP_391536). α-helices and β-sheets in the *B*. *subtilis* SpoIIQ extracellular domain (determined from the SpoIIQ-SpoIIIAH complex, [37,38]) are indicated as a helix or black arrow above the sequence alignment, respectively, with the first and last α-helices and β-sheets being labeled. Asterisks indicate *B*. *subtilis* SpoIIQ residues that directly interact with SpoIIIAH. The remaining SpoIIQs are from *B*. *cereus* serovar *anthracis* (YP_003794960), *C*. *botulinum* Loch Maree Type A3 (ACA54665), *C*. *acetobutylicum* ATCC 824 (NP_349463), *C*. *perfringens* str. 13 (BAB81888), *C*. *bartlettii* CAG 1329 (CDA09218), *C*. *bifermentans* ATCC 19299 (EQK48677), C. *sordellii* VPI 9048 (EPZ61518), and *C*. *difficile* 630 (YP_001086594). *C*. *bartlettii*, *C*. *bifermentans*, *C*. *sordellii* and *C*. *difficile* are part of the *Peptoclostridium* spp. [78].
(TIF)

S4 Fig Construction of *spoIIQ*, *spoIIIA*, and *spoIIIAH* mutants in *C*. *difficile*. (A) Schematic of *C*. *difficile spoIIIA* operon structure. The percent similarity between *C*. *difficile* and *B*. *subtilis* SpoIIIAA-AH proteins is shown. Bent arrows indicate promoters identified by Saujet *et al*. through global transcriptional start-site mapping [29]. (B) Schematic of the group II intron targeted gene disruption system. (C) Colony PCR analysis of *spoIIQ* ^*–*^, *spoIIIA* ^*–*^, and *spoIIIAH* ^*−*^strains compared to wild type (WT) using primers that flank the gene of interest. The group II intron insertion is ~2 kb.
(TIF)

S5 Fig Plasmid complementation rescues spore formation in *C*. *difficile spoIIQ* ^*–*^, *spoIIIA* ^*–*^, and *spoIIIAH* ^*−*^strains. Fluorescence microscopy of *spoIIQ* ^*−*^(*IIQ* ^*–*^), *spoIIIA* ^***−***^(*IIIA* ^*–*^), and *spoIIIAH* ^***−***^(*IIIAH* ^*–*^) complementation strains grown on sporulation media for 21 hrs using the lipophilic dye FM4-64 (red) and Hoechst nucleoid stain (blue). The strains carry empty vector (EV), or the *spoIIQ* (*IIQ*), or *spoIIIA* operon (*IIIA*), or *spoIIIAH* (*IIIAH*) complementation constructs. Yellow arrows designate forespores that have not completed engulfment, although they stain with Hoechst and FM4-64; blue arrows designate cells that have completed engulfment and stain with both Hoechst and FM4-64; green arrows designate forespore compartments that have completed engulfment and exclude Hoechst but stain with FM4-64; white arrows designate forespores that have completed engulfment and exclude Hoechst and FM4-64; pink arrows designate free spores. The efficiency of heat-resistant spore formation was determined for each strain relative to WT from three biological replicates. Scale bars represent 5 μm.
(TIF)

S6 Fig Analysis of *spoIIQ* ^*–*^, *spoIIIA* ^*–*^, and *spoIIIAH* ^*−*^complementation strains. (A) Western blot analyses of wildtype (WT), *spo0A* ^*-*^, *spoIIQ* ^*−*^(*IIQ* ^*–*^), *spoIIIA* ^***−***^(*IIIA* ^*–*^), or *spoIIIAH* ^***−***^(*IIIAH* ^*–*^) strains carrying empty vector (EV) or *spoIIQ* (*IIQ*), *spoIIIA* operon (*IIIA*), *spoIIIAH* (*IIIA*), or *spoIIIAH* (*IIIAH*) complementation constructs. Spo0A levels serve as a loading control for sporulation induction. (B) qRT-PCR analysis of *spoIIIAA* transcripts of wildtype, *spo0A* ^*–*^, or *spoIIIA* ^*−*^(*IIIA* ^*–*^) carrying empty vector (EV), K167A *spoIIIA* operon (K167A), or *spoIIIA* operon (*IIIA*) complementation constructs. Transcript levels were calculated relative to the *spo0A* ^*–*^ strain after normalization to the housekeeping gene *rpoB* using the standard curve method. Error bars indicate the standard error of the mean. Data represents the average of three biological replicates. Error bars indicate the standard error of the mean. Statistically significant changes in transcript levels were determined relative to WT and are represented by adjusted p-values determined by a one-way ANOVA and Dunnett’s test. *p < 0.05. n.a. indicates not applicable since the region amplified is downstream of the disrupted *spoIIIAA* gene.
(TIF)

S7 Fig Variable sporulation phenotypes of *C*. *difficile spoIIQ* ^*–*^, *spoIIIA* ^*–*^, and *spoIIIAH* ^*−*^strains. Transmission electron microscopy (TEM) of *spoIIQ* ^*–*^, *spoIIIA* ^***–***^, and *spoIIIAH* ^***−***^grown for 24 hrs on sporulation media. (A) Rare example of *spoIIQ* ^*−*^ *and spoIIIA* ^***−***^cells that have completed engulfment (green arrows). Black arrows designate coat localized around the forespore compartment. The *spoIIIAH* mutant was not observed to complete engulfment. (B) The forespore regions of *spoIIQ* ^*–*^, *spoIIIA* ^*–*^, and *spoIIIAH* ^*−*^cells exhibiting forespore collapse (blue arrows), which occurred in 13%, 14%, and 27% of cells, respectively. Scale bars represent 500 nm.
(TIF)

S8 Fig σG activity is unaffected in spoIIQ, spoIIIA, and spoIIIAH mutants. Transcript levels of the σ^G^ regulon genes *spoVT*, *CD1430*, and *spoVAD* in wild type (WT), *spo0A* ^*–*^, *sigF* ^*–*^, *spoIIQ* ^*−*^(*IIQ* ^*–*^), *sigE* ^*–*^, *spoIIIA* ^*−*^(*IIIA* ^*–*^), *spoIIIAH* ^*−*^(*IIIAH* ^*–*^), and *sigG* ^*−*^induced to sporulate for 25 hrs as measured by qRT-PCR. Transcript levels were calculated relative to the *spo0A* ^*–*^ strain after normalization to the housekeeping gene *rpoB* using the standard curve method. Data represents the average of three biological replicates. Error bars indicate the standard error of the mean. Statistically significant changes in transcript levels were determined relative to WT and are represented by adjusted p-values determined by a one-way ANOVA and Dunnett’s test. ***p < 0.0005, **p < 0.01, *p < 0.05.
(TIF)

S9 Fig Mutation of the SpoIIQ LytM catalytic histidine 120 leads to a defect in spore formation. TEM analyses of wildtype (WT) and *spoIIQ* ^*−*^(*IIQ* ^*–*^) strains carrying empty vector (EV), the *spoIIQ* H120A LytM mutation (H120A) complementation construct, and the wildtype *spoIIQ* complementation construct (*IIQ*). The forespore region of these strains is shown on the right. Black arrows indicate regions that resemble coat layers surrounding the forespore. White arrows indicate coat that appears anchored to the leading edge of the engulfing membrane but is not intimately associated with the mother cell-forespore interface. Yellow arrows demarcate coat that has mislocalized to the cytosol. Scale bars represent 500 nm. The efficiency of heat-resistant (HR) spore formation was determined for each strain relative to WT across four biological replicates. Engulfment complete (EC) cells designates the number of cells in the population that completed engulfment out of at least 50 sporulating cells that had initiated engulfment or progressed beyond. Two representative phenotypes for the H120A mutant are shown; A designates engulfment complete, B designates engulfment incomplete.
(TIF)

S10 Fig The Walker A motif is critical for spore formation. Wild type carrying empty vector (WT/EV) and *spoIIIA* ^*−*^(*IIIA* ^*–*^) strains carrying either empty vector (EV), the *IIIA* K167A complementation construct (K167A), or wildtype *IIIA* complementation construct (*IIIA*) were grown on sporulation media for 22 hrs and evaluated by live differential interference contrast (DIC) and fluorescence microscopy using the Hoechst nucleoid stain (blue) and lipophilic dye FM4-64 (red). Yellow arrows designate forespores that have not completed engulfment, although they stain with Hoechst and FM4-64; green arrows designate forespore compartments that have completed engulfment and exclude Hoechst but stain with FM4-64; white arrows designate forespores that have completed engulfment and exclude Hoechst and FM4-64; pink arrows designate free spores. Scale bars represent 5 μm.
(TIF)

S11 Fig Timecourse of peptidoglycan labeling of sporulating cells. Cells induced to sporulate were incubated with alkDala and analyzed over the course of 40 minutes at 10 minute intervals. Sporulating cells were surveyed for sporulation based on DIC, Hoechst incorporation, and alkDala labeling (Shown in Fig 8). alkDala incorporation was scored based on no incorporation at the forespore compartment (black bars), labeling of a polar septum (designating a cell undergoing asymmetric division, gray bars), labeling at the forespore side of the forespore compartment (yellow bars), labeling of the middle section of the forespore compartment (red bars), labeling at the mother cell side of the forespore compartment (blue bars), and full labeling of the spore (green bars). At least 50 cells per time point were analyzed.
(TIF)

S12 Fig Fluorescent labeling of peptidoglycan transformations during cell division and sporulation of wildtype, *spoIIQ* ^*–*^, and *sigE* ^*−*^strains. (A) Examples of cells undergoing vegetative cell division (yellow arrows). Strains were induced to sporulate on solid media for 14 hrs then resuspended in liquid sporulation media. Alkyne D-alanine (alkDala) or D-alanine (background control shown in (B)) was incubated with the cells for 30’. After fixation, permeabilization, and copper-catalyzed cycloaddition of an azide-conjugated fluorophore, Hoechst nucleoid dye was added, and cells were visualized by light microscopy. Scale bars represent 2 μm.
(TIF)

S13 Fig Imipenem inhibition of peptidoglycan transformations in *C*. *difficile*. AlkDala incorporation during peptidoglycan transformations after treatment with cell wall inhibitors vancomycin and imipenem was evaluated by flow cytometry. Mean fluorescence intensities (MFIs) were determined for WT cells incubated with Dala or alkDala after treatment with 2X MIC determined for vancomycin or imipenem or no treatment controls. MFIs are based on three biological replicates, and statistically significant changes were determined by an ordinary one-way ANOVA and Tukey’s test. ****p < 0.0001, ns = no statistical difference.
(TIF)

S1 Table *E*. *coli* strains used in this study. (DOCX)

S2 Table Plasmids used in this study. (DOCX)

S3 Table Primers used in this study. (DOCX)
