# Supplementary figures and images for: Regulation of Clostridium difficile Spore Formation by the SpoIIQ and SpoIIIA Proteins
Source: PLoS Genet. 2015 Oct 14;11(10):e1005562. doi: 10.1371/journal.pgen.1005562 (PMC4605598; doi:10.1371/journal.pgen.1005562)

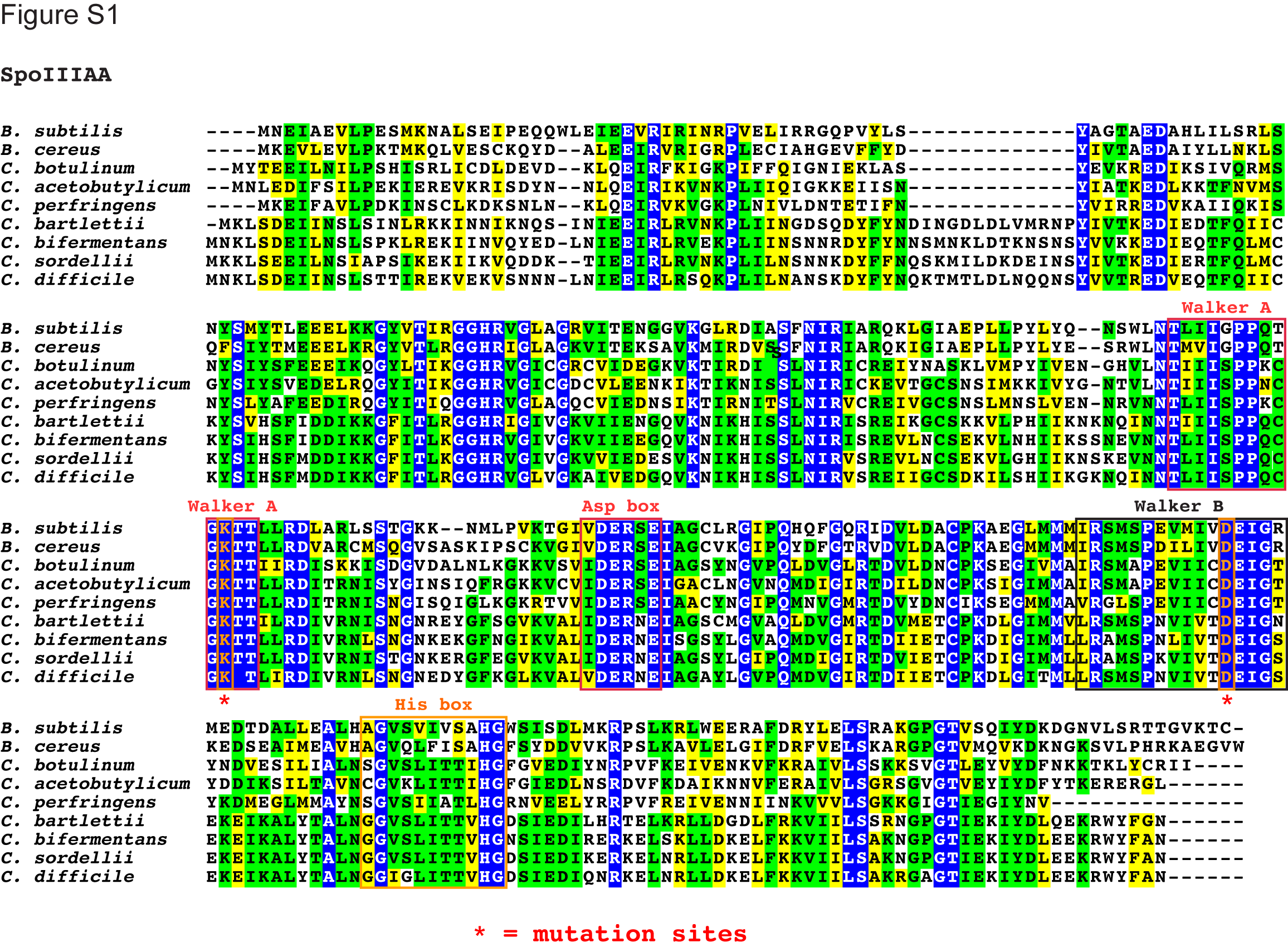

Supplement: S1 Fig — Completely conserved, identical residues are blocked in blue, conserved identical residues are blocked in green, and conserved similar residues in yellow. The conserved motifs (boxes) found in all secretion NTPases are highlighted [20]. The conserved lysine in the Walker A motif that was mutated (K167 in C. difficile SpoIIIAH) and conserved aspartate in the Walker B motif (D244 in C. difficile SpoIIIAH) are shown boxed in orange. SpoIIIAA sequences are from B. subtilis serovar subtilis str. 168 (CAA43959) B. cereus serovar anthracis (YP_003793897), C. botulinum ATCC 3502 (CAL83436), C. acetobutylicum ATCC 824 (AE007711_9), C. perfringens str. 13 (NP_562749), C. bartlettii CAG 1329 (WP_022072529), C. bifermentans ATCC 19299 (EQK45222), C. sordellii VPI 9048 (EPZ57018), and C. difficile 630 (YP_001087685). C. bartlettii, C. bifermentans, C. sordellii and C. difficile are part of the Peptoclostridium spp. [78]. (TIF) [file pgen.1005562.s001.tif]

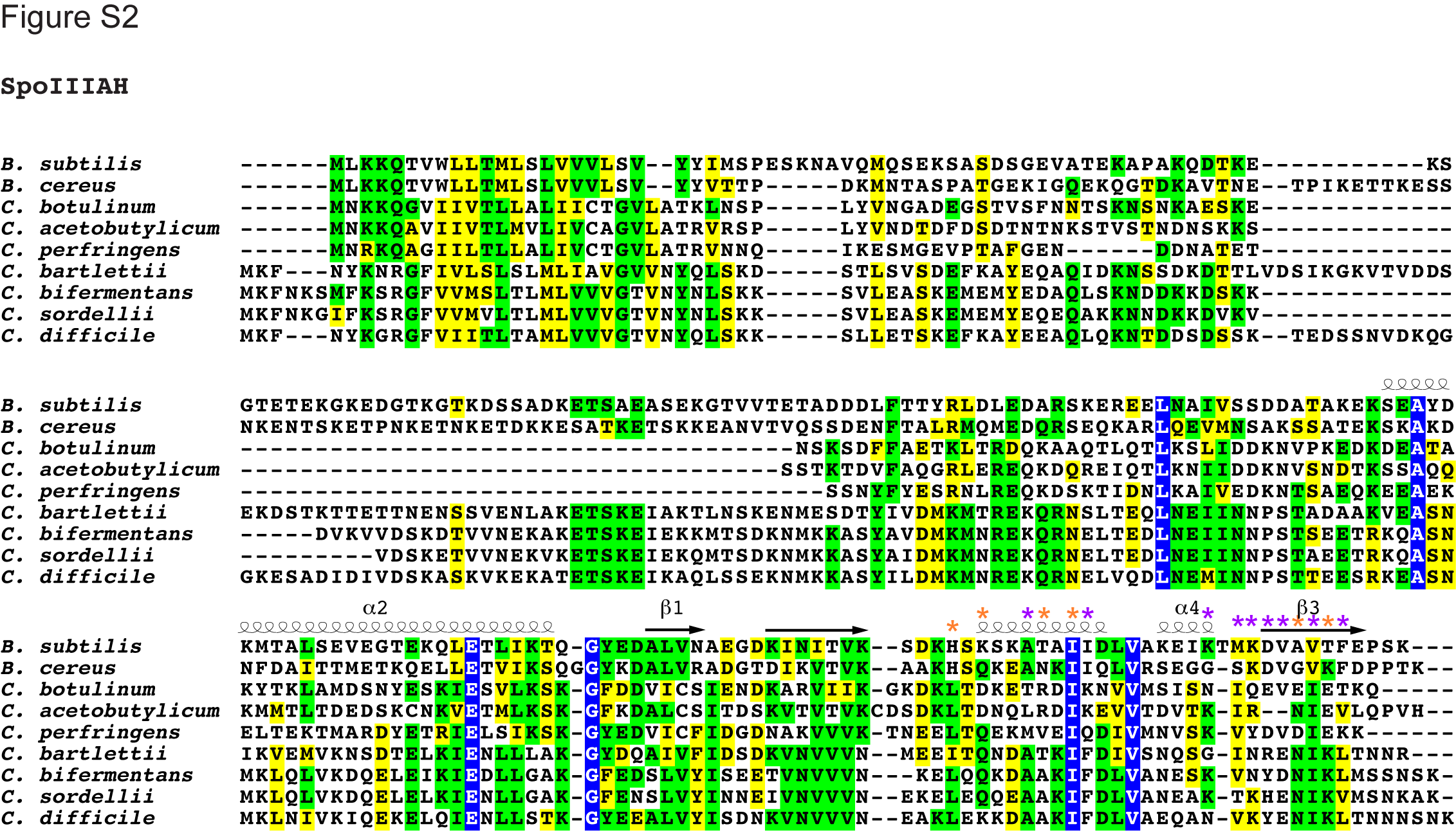

Supplement: S2 Fig — Completely conserved, identical residues are blocked in blue, conserved identical residues are blocked in green, and conserved similar residues in yellow. α-helices and β-sheets in the B. subtilis SpoIIIAH (NP_390316) extracellular domain (determined from the SpoIIQ-SpoIIIAH complex, [37,38]) are indicated as a helix or black arrow above the sequence alignment, respectively, with the first and last α-helices and β-sheets being labeled. Purple asterisks identify B. subtilis SpoIIIAH residues that directly interact with SpoIIQ as determined by both Levdikov et al. and Meisner et al. [37,38]. Orange asterisks indicate B. subtilis SpoIIIAH residues that directly interact with SpoIIQ as determined by Levdikov et al. [37]. The remaining sequences are from B. cereus serovar anthracis (YP_003793890), C. botulinum ATCC 3502 (YP_001254390), C. acetobutylicum ATCC 824 (AE007711_2), C. perfringens str. 13 (NP_562742), C. bartlettii CAG 128 (CDA09218), C. bifermentans ATCC 19299 (EQK45176), C. sordellii VPI 9048 (EPZ57011), and C. difficile 630 (YP_001087692). C. bartlettii, C. bifermentans, C. sordellii and C. difficile are part of the Peptoclostridium genus [78]. (TIF) [file pgen.1005562.s002.tif]

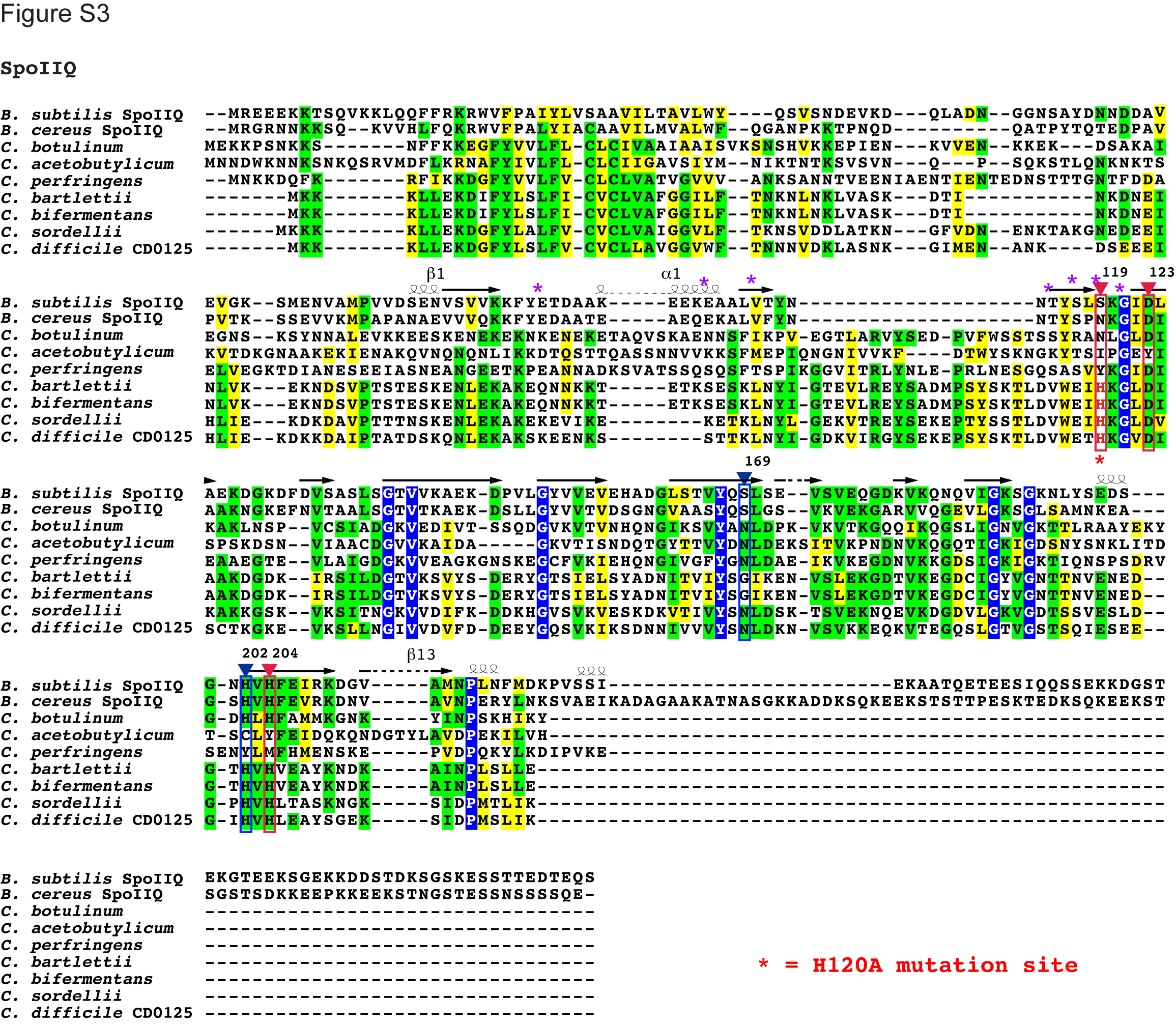

Supplement: S3 Fig — Orthologs were identified based on a Hidden Markov Model search using HHMER [79]. Completely conserved, identical residues are blocked in blue, conserved identical residues are blocked in green, and conserved similar residues in yellow. A red triangle demarcates active site residues (boxed in red), with phosphate binding residues in the active site being marked with a blue triangle (boxed in blue) [32]. The intact active site His in the HxxxD motif of the metallopeptidase found in C. difficile CD0125 is shown in red; the active site His has been mutated to Ser in B. subtilis SpoIIQ. Residue numbering is based on B. subtilis SpoIIQ (NP_391536). α-helices and β-sheets in the B. subtilis SpoIIQ extracellular domain (determined from the SpoIIQ-SpoIIIAH complex, [37,38]) are indicated as a helix or black arrow above the sequence alignment, respectively, with the first and last α-helices and β-sheets being labeled. Asterisks indicate B. subtilis SpoIIQ residues that directly interact with SpoIIIAH. The remaining SpoIIQs are from B. cereus serovar anthracis (YP_003794960), C. botulinum Loch Maree Type A3 (ACA54665), C. acetobutylicum ATCC 824 (NP_349463), C. perfringens str. 13 (BAB81888), C. bartlettii CAG 1329 (CDA09218), C. bifermentans ATCC 19299 (EQK48677), C. sordellii VPI 9048 (EPZ61518), and C. difficile 630 (YP_001086594). C. bartlettii, C. bifermentans, C. sordellii and C. difficile are part of the Peptoclostridium spp. [78]. (TIF) [file pgen.1005562.s003.tif]

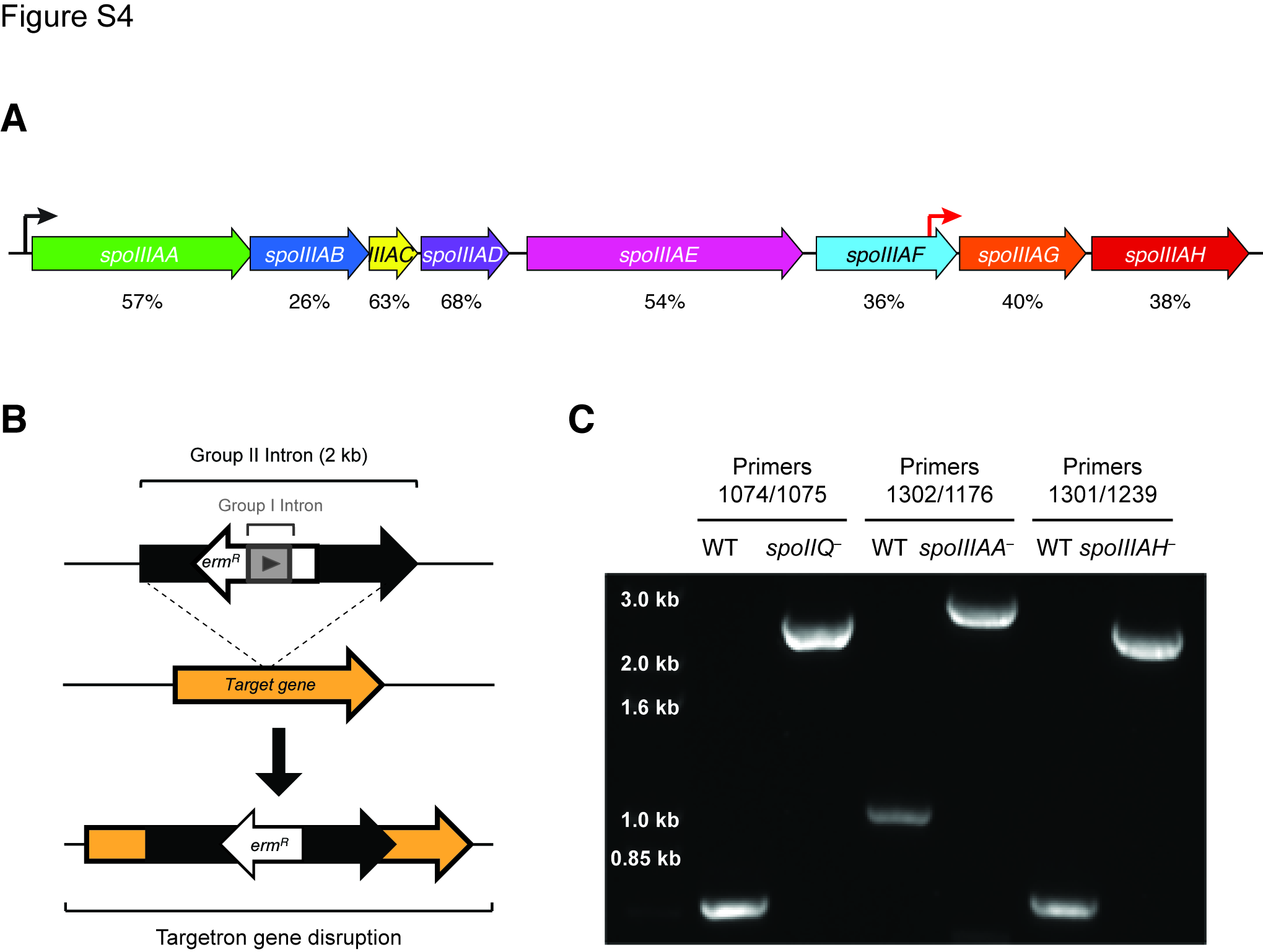

Supplement: S4 Fig — (A) Schematic of C. difficile spoIIIA operon structure. The percent similarity between C. difficile and B. subtilis SpoIIIAA-AH proteins is shown. Bent arrows indicate promoters identified by Saujet et al. through global transcriptional start-site mapping [29]. (B) Schematic of the group II intron targeted gene disruption system. (C) Colony PCR analysis of spoIIQ –, spoIIIA –, and spoIIIAH −strains compared to wild type (WT) using primers that flank the gene of interest. The group II intron insertion is ~2 kb. (TIF) [file pgen.1005562.s004.tif]

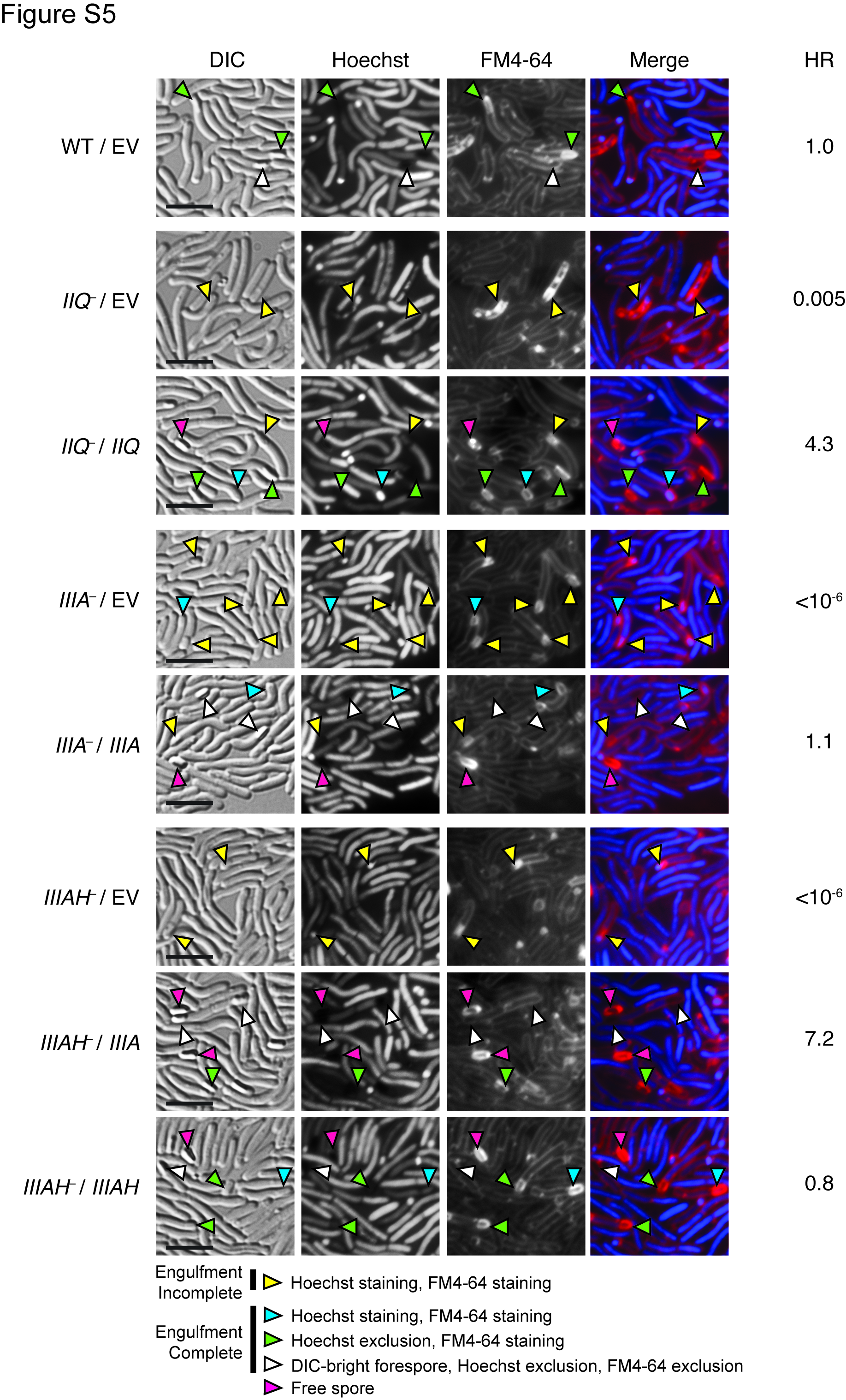

Supplement: S5 Fig — Fluorescence microscopy of spoIIQ −(IIQ –), spoIIIA −(IIIA –), and spoIIIAH −(IIIAH –) complementation strains grown on sporulation media for 21 hrs using the lipophilic dye FM4-64 (red) and Hoechst nucleoid stain (blue). The strains carry empty vector (EV), or the spoIIQ (IIQ), or spoIIIA operon (IIIA), or spoIIIAH (IIIAH) complementation constructs. Yellow arrows designate forespores that have not completed engulfment, although they stain with Hoechst and FM4-64; blue arrows designate cells that have completed engulfment and stain with both Hoechst and FM4-64; green arrows designate forespore compartments that have completed engulfment and exclude Hoechst but stain with FM4-64; white arrows designate forespores that have completed engulfment and exclude Hoechst and FM4-64; pink arrows designate free spores. The efficiency of heat-resistant spore formation was determined for each strain relative to WT from three biological replicates. Scale bars represent 5 μm. (TIF) [file pgen.1005562.s005.tif]

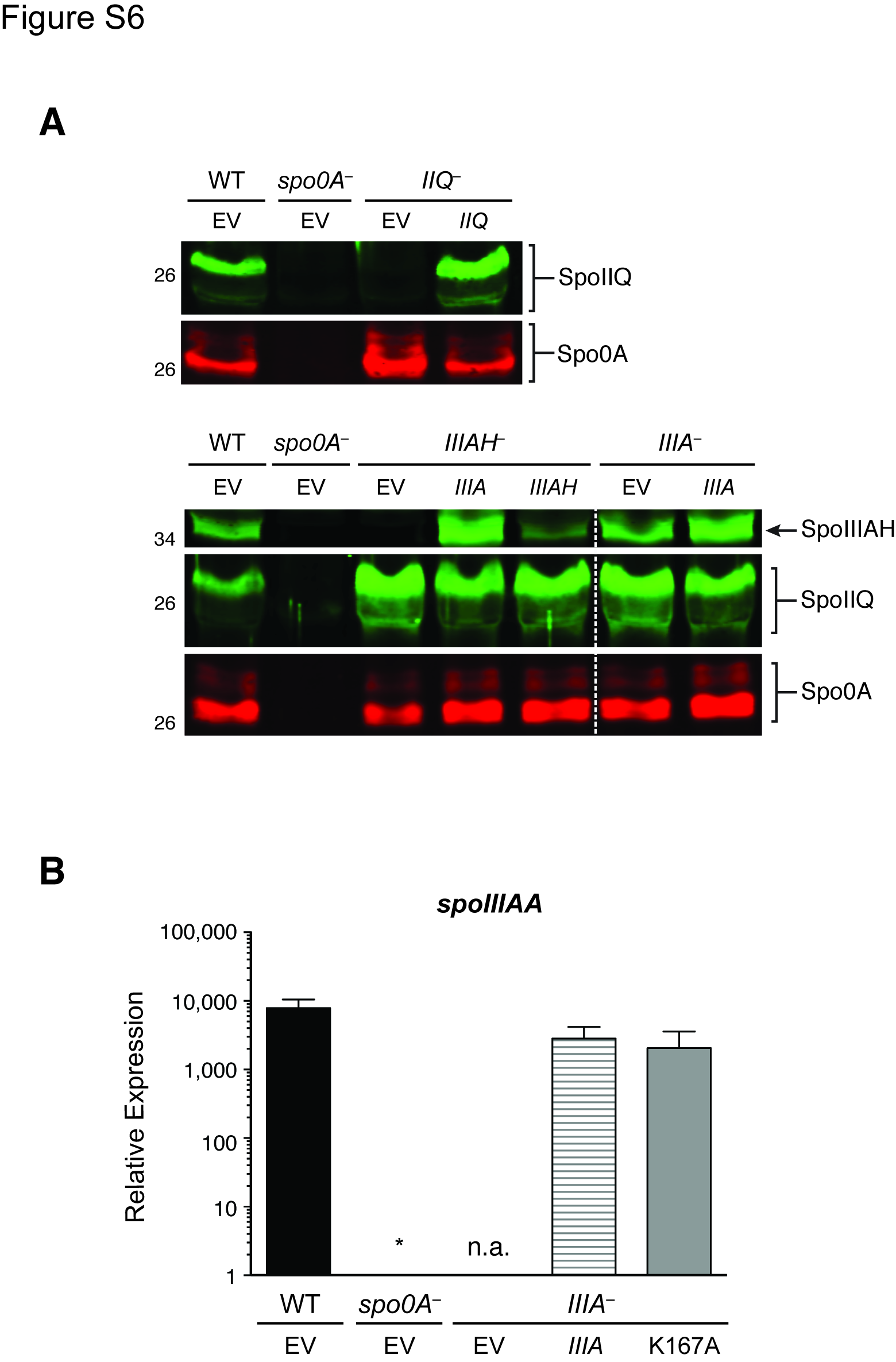

Supplement: S6 Fig — (A) Western blot analyses of wildtype (WT), spo0A -, spoIIQ −(IIQ –), spoIIIA −(IIIA –), or spoIIIAH −(IIIAH –) strains carrying empty vector (EV) or spoIIQ (IIQ), spoIIIA operon (IIIA), spoIIIAH (IIIA), or spoIIIAH (IIIAH) complementation constructs. Spo0A levels serve as a loading control for sporulation induction. (B) qRT-PCR analysis of spoIIIAA transcripts of wildtype, spo0A –, or spoIIIA −(IIIA –) carrying empty vector (EV), K167A spoIIIA operon (K167A), or spoIIIA operon (IIIA) complementation constructs. Transcript levels were calculated relative to the spo0A – strain after normalization to the housekeeping gene rpoB using the standard curve method. Error bars indicate the standard error of the mean. Data represents the average of three biological replicates. Error bars indicate the standard error of the mean. Statistically significant changes in transcript levels were determined relative to WT and are represented by adjusted p-values determined by a one-way ANOVA and Dunnett’s test. *p < 0.05. n.a. indicates not applicable since the region amplified is downstream of the disrupted spoIIIAA gene. (TIF) [file pgen.1005562.s006.tif]

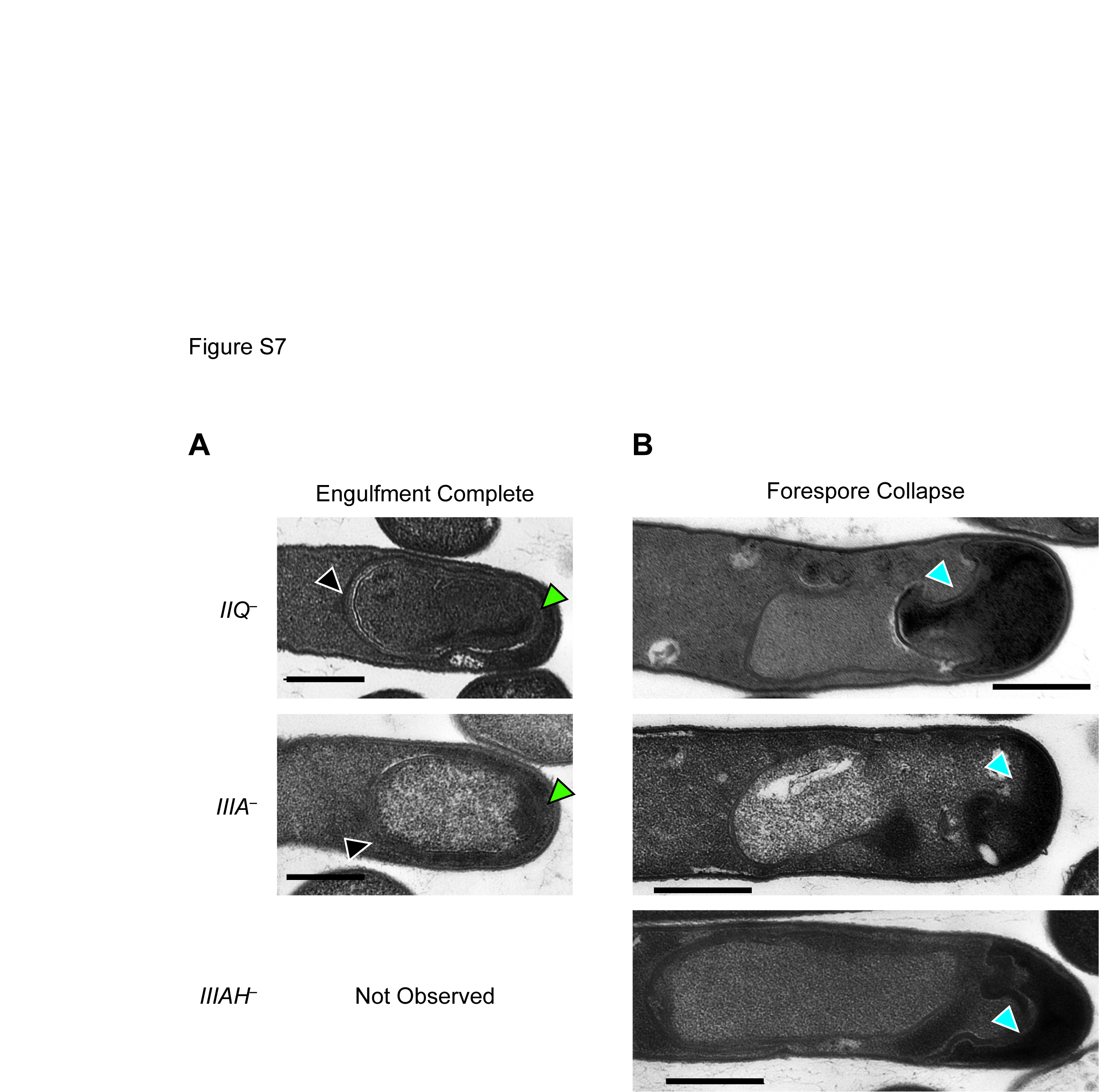

Supplement: S7 Fig — Transmission electron microscopy (TEM) of spoIIQ –, spoIIIA –, and spoIIIAH −grown for 24 hrs on sporulation media. (A) Rare example of spoIIQ − and spoIIIA −cells that have completed engulfment (green arrows). Black arrows designate coat localized around the forespore compartment. The spoIIIAH mutant was not observed to complete engulfment. (B) The forespore regions of spoIIQ –, spoIIIA –, and spoIIIAH −cells exhibiting forespore collapse (blue arrows), which occurred in 13%, 14%, and 27% of cells, respectively. Scale bars represent 500 nm. (TIF) [file pgen.1005562.s007.tif]

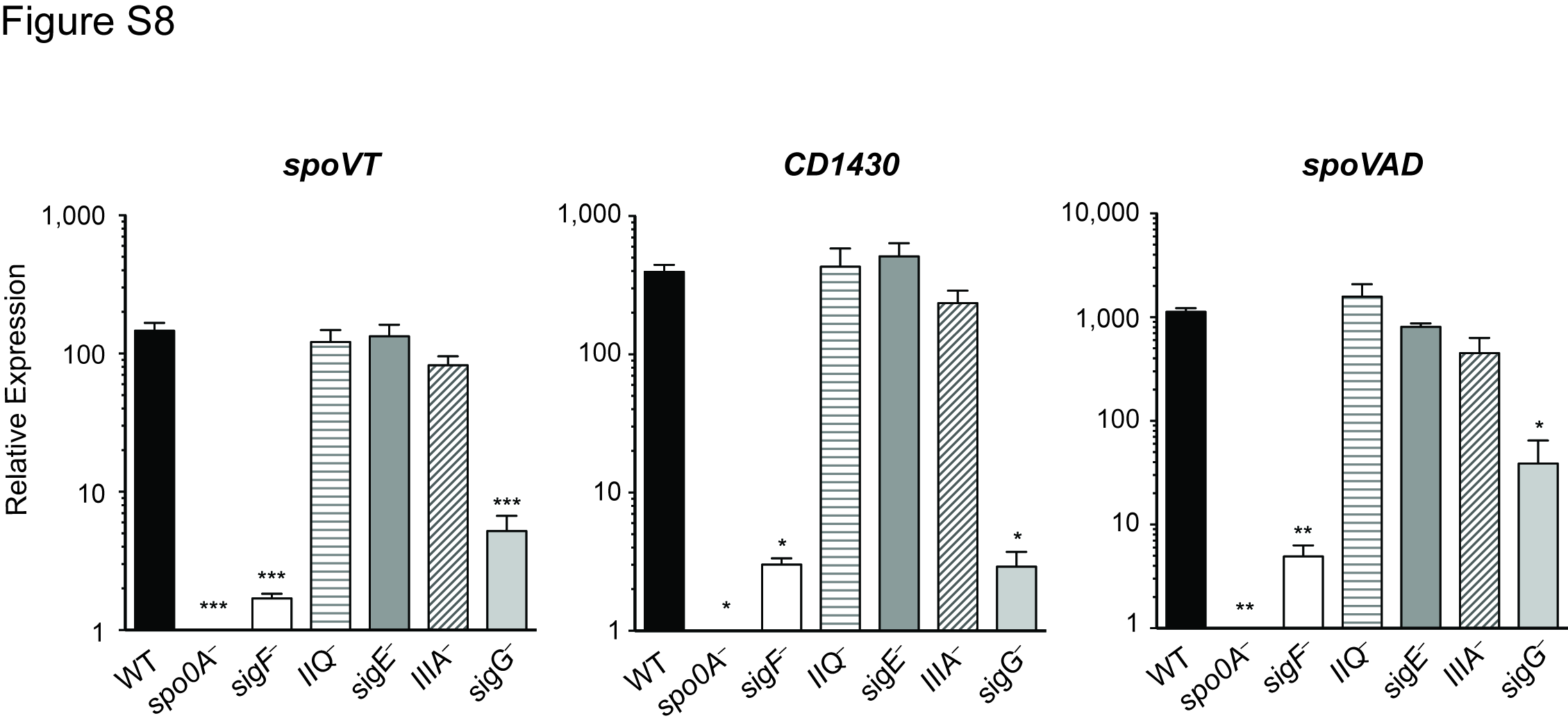

Supplement: S8 Fig — Transcript levels of the σG regulon genes spoVT, CD1430, and spoVAD in wild type (WT), spo0A –, sigF –, spoIIQ −(IIQ –), sigE –, spoIIIA −(IIIA –), spoIIIAH −(IIIAH –), and sigG −induced to sporulate for 25 hrs as measured by qRT-PCR. Transcript levels were calculated relative to the spo0A – strain after normalization to the housekeeping gene rpoB using the standard curve method. Data represents the average of three biological replicates. Error bars indicate the standard error of the mean. Statistically significant changes in transcript levels were determined relative to WT and are represented by adjusted p-values determined by a one-way ANOVA and Dunnett’s test. ***p < 0.0005, **p < 0.01, *p < 0.05. (TIF) [file pgen.1005562.s008.tif]

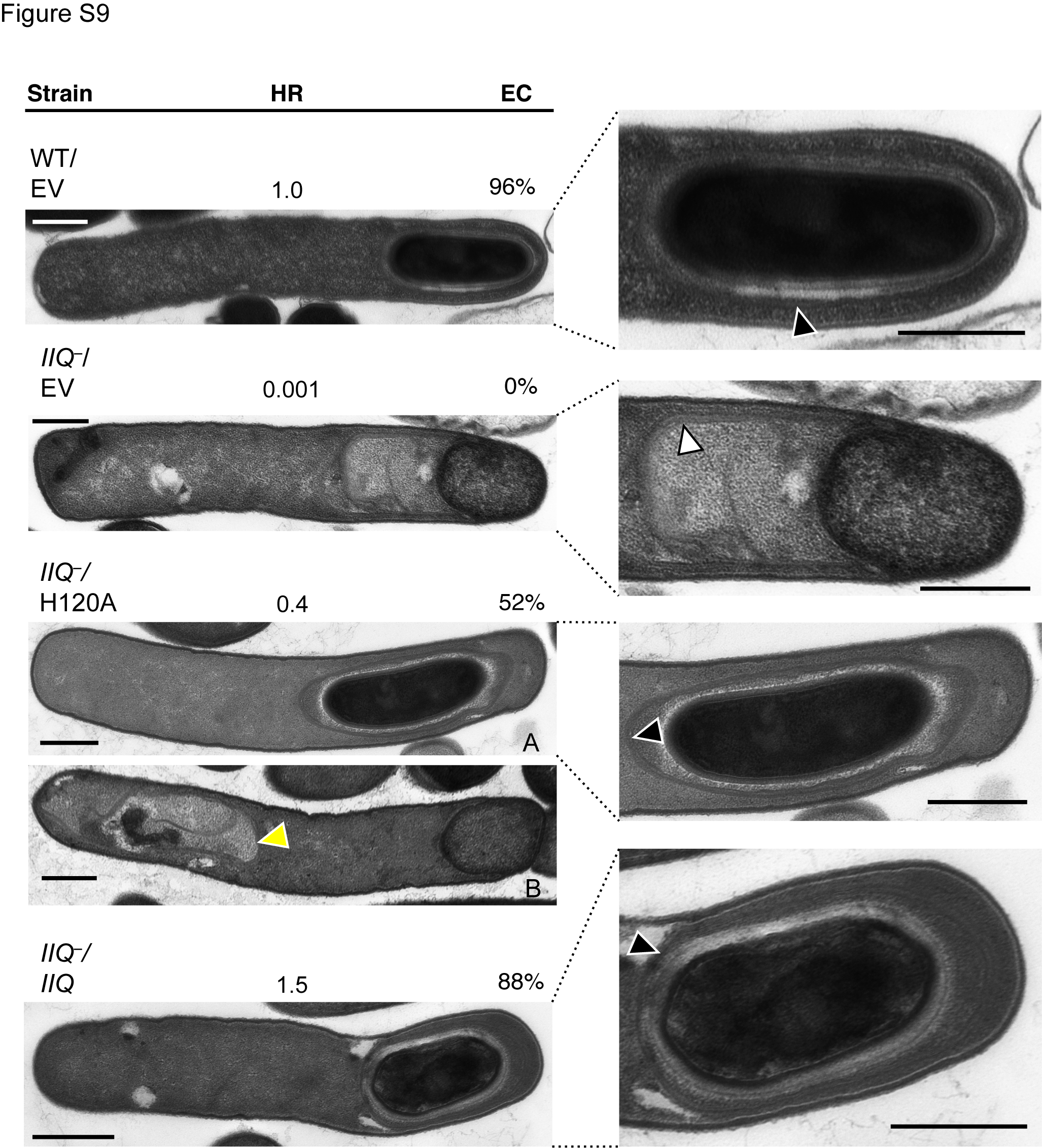

Supplement: S9 Fig — TEM analyses of wildtype (WT) and spoIIQ −(IIQ –) strains carrying empty vector (EV), the spoIIQ H120A LytM mutation (H120A) complementation construct, and the wildtype spoIIQ complementation construct (IIQ). The forespore region of these strains is shown on the right. Black arrows indicate regions that resemble coat layers surrounding the forespore. White arrows indicate coat that appears anchored to the leading edge of the engulfing membrane but is not intimately associated with the mother cell-forespore interface. Yellow arrows demarcate coat that has mislocalized to the cytosol. Scale bars represent 500 nm. The efficiency of heat-resistant (HR) spore formation was determined for each strain relative to WT across four biological replicates. Engulfment complete (EC) cells designates the number of cells in the population that completed engulfment out of at least 50 sporulating cells that had initiated engulfment or progressed beyond. Two representative phenotypes for the H120A mutant are shown; A designates engulfment complete, B designates engulfment incomplete. (TIF) [file pgen.1005562.s009.tif]

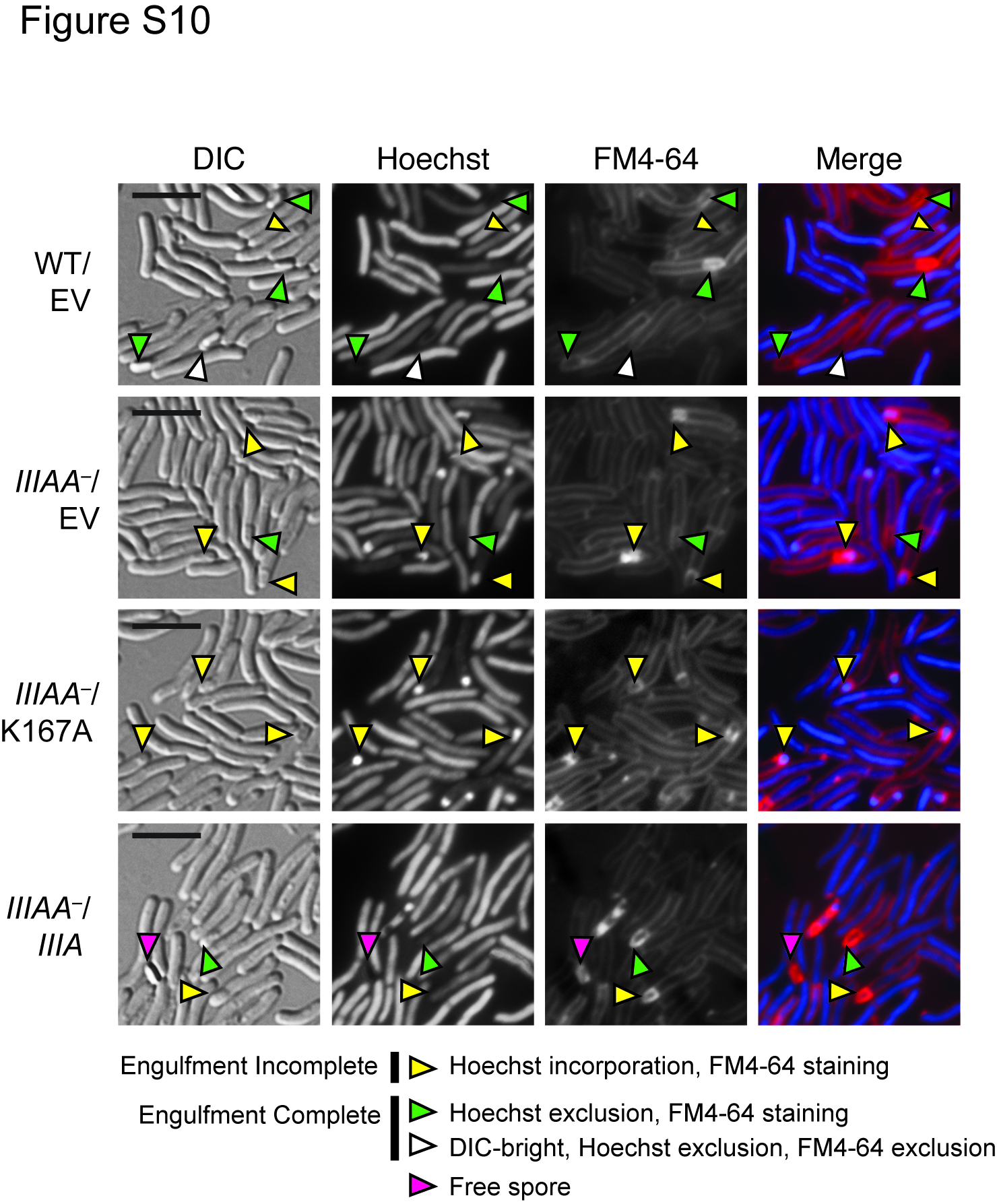

Supplement: S10 Fig — Wild type carrying empty vector (WT/EV) and spoIIIA −(IIIA –) strains carrying either empty vector (EV), the IIIA K167A complementation construct (K167A), or wildtype IIIA complementation construct (IIIA) were grown on sporulation media for 22 hrs and evaluated by live differential interference contrast (DIC) and fluorescence microscopy using the Hoechst nucleoid stain (blue) and lipophilic dye FM4-64 (red). Yellow arrows designate forespores that have not completed engulfment, although they stain with Hoechst and FM4-64; green arrows designate forespore compartments that have completed engulfment and exclude Hoechst but stain with FM4-64; white arrows designate forespores that have completed engulfment and exclude Hoechst and FM4-64; pink arrows designate free spores. Scale bars represent 5 μm. (TIF) [file pgen.1005562.s010.tif]

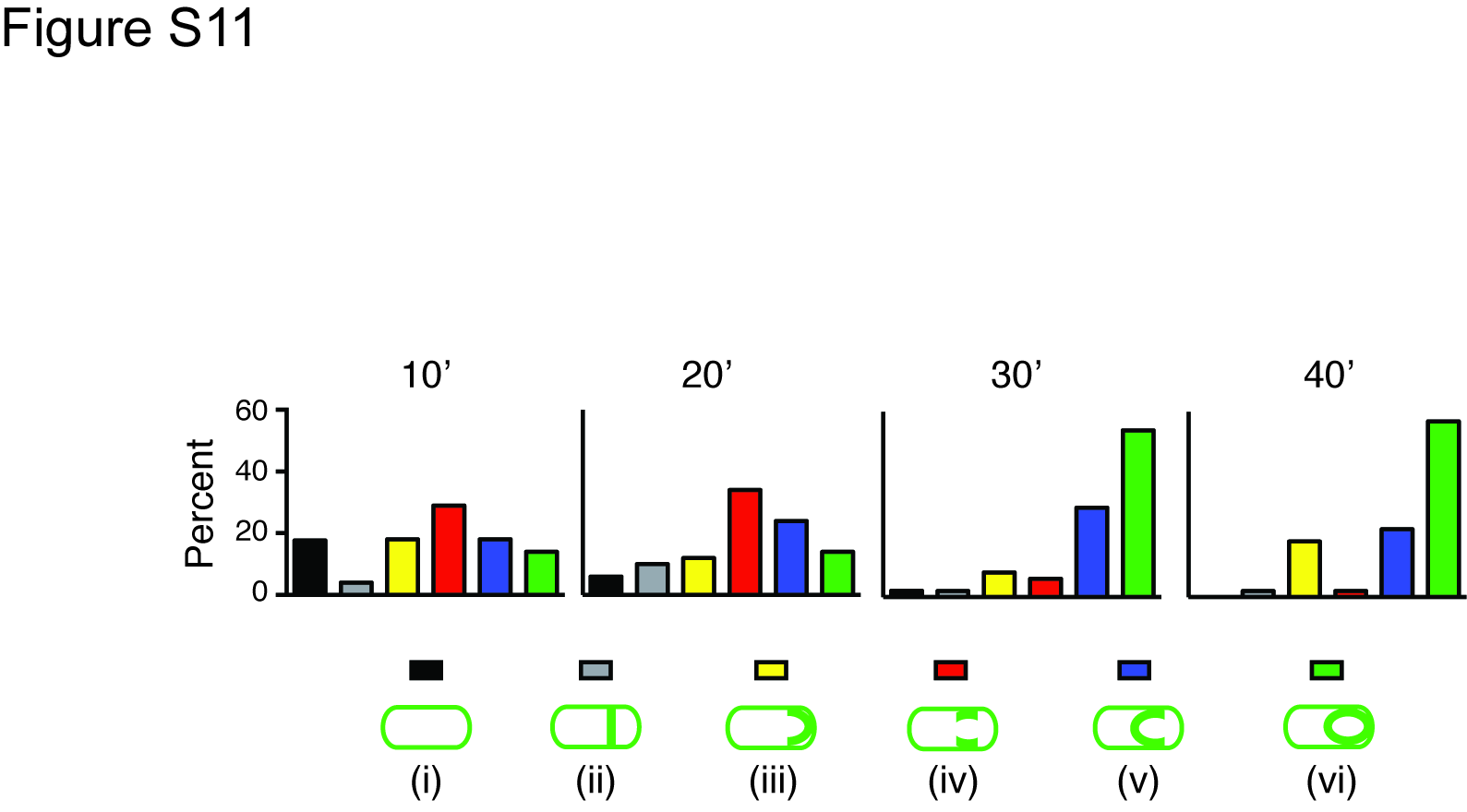

Supplement: S11 Fig — Cells induced to sporulate were incubated with alkDala and analyzed over the course of 40 minutes at 10 minute intervals. Sporulating cells were surveyed for sporulation based on DIC, Hoechst incorporation, and alkDala labeling (Shown in Fig 8). alkDala incorporation was scored based on no incorporation at the forespore compartment (black bars), labeling of a polar septum (designating a cell undergoing asymmetric division, gray bars), labeling at the forespore side of the forespore compartment (yellow bars), labeling of the middle section of the forespore compartment (red bars), labeling at the mother cell side of the forespore compartment (blue bars), and full labeling of the spore (green bars). At least 50 cells per time point were analyzed. (TIF) [file pgen.1005562.s011.tif]

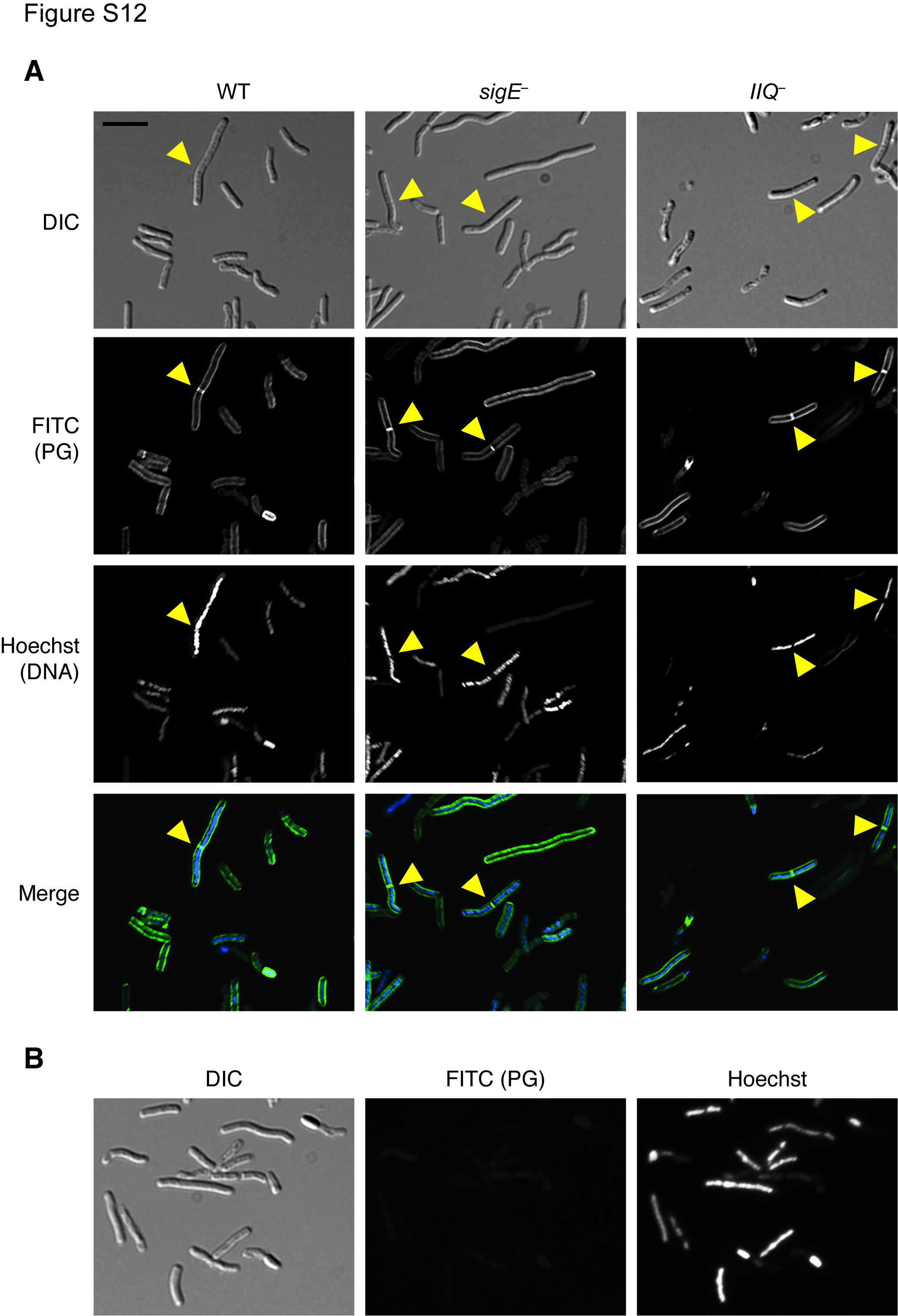

Supplement: S12 Fig — (A) Examples of cells undergoing vegetative cell division (yellow arrows). Strains were induced to sporulate on solid media for 14 hrs then resuspended in liquid sporulation media. Alkyne D-alanine (alkDala) or D-alanine (background control shown in (B)) was incubated with the cells for 30’. After fixation, permeabilization, and copper-catalyzed cycloaddition of an azide-conjugated fluorophore, Hoechst nucleoid dye was added, and cells were visualized by light microscopy. Scale bars represent 2 μm. (TIF) [file pgen.1005562.s012.tif]

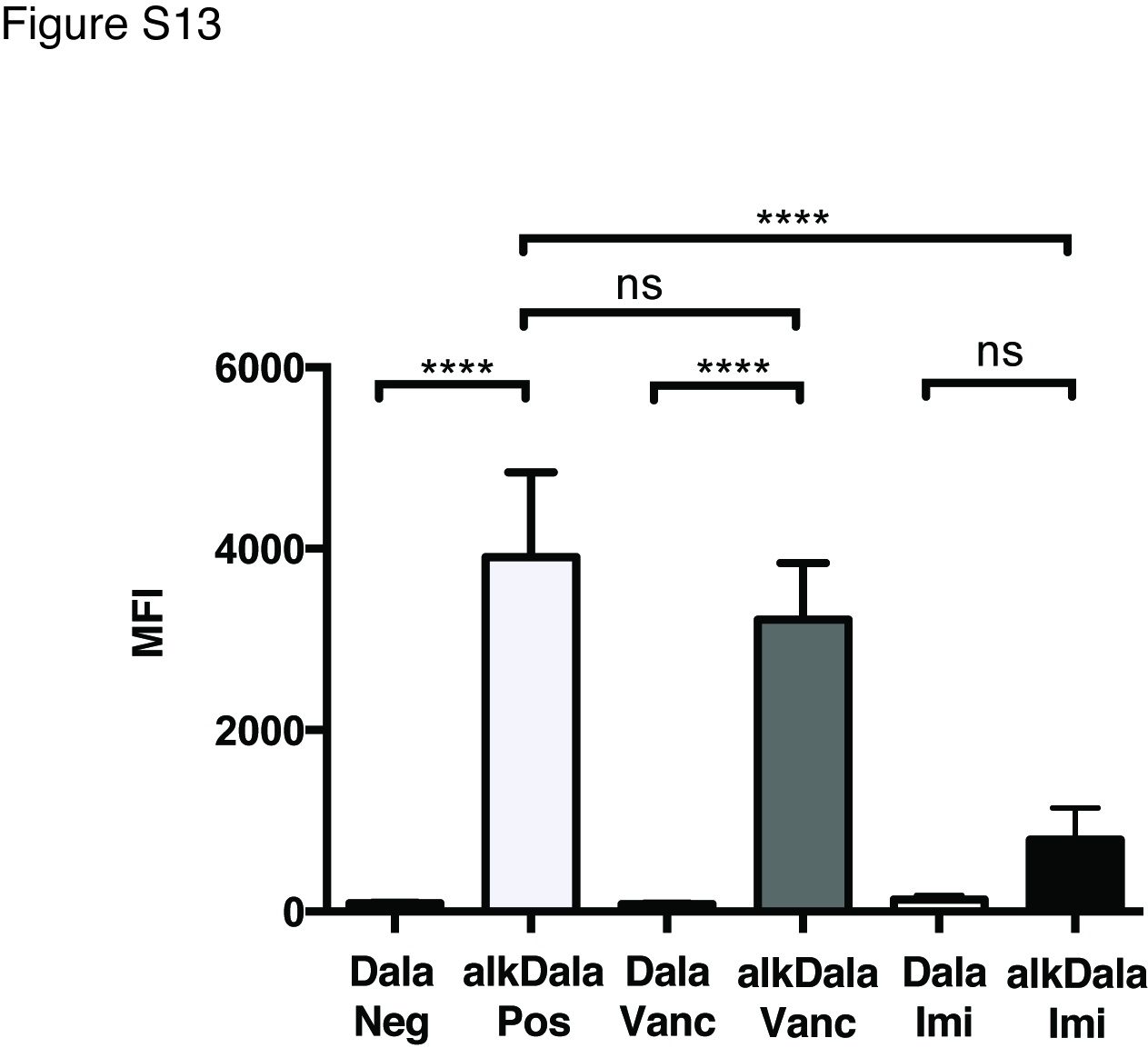

Supplement: S13 Fig — AlkDala incorporation during peptidoglycan transformations after treatment with cell wall inhibitors vancomycin and imipenem was evaluated by flow cytometry. Mean fluorescence intensities (MFIs) were determined for WT cells incubated with Dala or alkDala after treatment with 2X MIC determined for vancomycin or imipenem or no treatment controls. MFIs are based on three biological replicates, and statistically significant changes were determined by an ordinary one-way ANOVA and Tukey’s test. ****p < 0.0001, ns = no statistical difference. (TIF) [file pgen.1005562.s013.tif]
