# Supplementary material for: Regulation of Clostridium difficile Spore Formation by the SpoIIQ and SpoIIIA Proteins
Source: PLoS Genet. 2015 Oct 14;11(10):e1005562. doi: 10.1371/journal.pgen.1005562 (PMC4605598; doi:10.1371/journal.pgen.1005562)
Supplement: S1 Table — (DOCX) [file pgen.1005562.s014.docx]

**Table S1. *E. coli* strains used in this study.**

| **Strain** | **Relevant genotype or features** | **Source or reference** |
| --- | --- | --- |
| DH5α | F– Φ80*lacZ*ΔM15 Δ(*lacZYA-argF*) U169 *recA1 endA1 hsdR17* (rK–, mK+) *phoA supE44* λ– *thi-1 gyrA96 relA1* | D. Cameron |
| BL21(DE3) | F– *ompT hsdSB*(rB–, mB–) *gal dcm* (DE3) | Novagen |
| HB101 | F- *mcrB mrr hsdS20*(rB- mB-) *recA13 leuB6 ara-13 proA2 lavYI galK2 xyl-6 mtl-1 rpsL20* | C. Ellermeier |
| 7 | pET22b in DH5α | D. Higgins |
| 269 | pET28a in DH5α | M. Bogyo |
| 556 | pJS107 in DH5α | J. Sorg |
| 655 | pMTL83151 in DH5α | This study |
| 686 | pMTL83151 in HB101/pK424 | This study |
| 701 | pMTL84151 in DH5α | This study |
| 703 | pMTL84151 in HB101/pK424 | This study |
| 908 | pJS107-*spoIIIAA* targeting bp 166 in HB101/pK424 | This study |
| 909 | pJS107-*spoIIQ* targeting bp 456 in HB101/pK424 | This study |
| 1058 | pMTL83151-*spoIIQ* in HB101/pK424 | This study |
| 1080 | pMTL83151-*spoIIIA* in HB101/pK424 | This study |
| 1197 | pJS107-*spoIIIAH* targeting bp 75 in HB101/pK424 | This study |
| 1301 | pET22b-*spoIIQ* 91-669 (*E. coli* codon optimized) in BL21(DE3) | This study |
| 1302 | pET22b-*spoIIIAH* 100-691 (*E. coli* codon optimized) in BL21(DE3) | This study |
| 1303 | pMTL83151-*spoIIIA* operon K167A in HB101/pK424 | This study |
| 1370 | pMTL84121-CotE-SNAP in HB101/pK424 | A. Henriques |
| 1378 | pET28a-HA-SpoIIIAH in BL21(DE3) | This study |
| 1380 | pMTL83151-P*_spoIIIAA_-spoIIIAH* in HB101/pK424 | This study |
| 1385 | pET22b + pET28a-HA-SpoIIIAH in BL21(DE3) | This study |
| 1386 | pET22b-His-SpoIIQ + pET28a-HA-SpoIIIAH in BL21(DE3) | This study |
| 1387 | pET22b-His-SpoIIQ + pET28aEV in BL21(DE3) | This study |
| 1404 | pET28a-HA-SpoVT in BL21(DE3) | This study |
| 1405 | pET22b-His-SpoIIQ + pET28a-HA-SpoVT in BL21(DE3) | This study |
| 1433 | pMTL84151-P*_sspA_*-SNAP in HB101/pK424 | This study |
