# Supplementary material for: Regulation of Clostridium difficile Spore Formation by the SpoIIQ and SpoIIIA Proteins
Source: PLoS Genet. 2015 Oct 14;11(10):e1005562. doi: 10.1371/journal.pgen.1005562 (PMC4605598; doi:10.1371/journal.pgen.1005562)
Supplement: S2 Table — (DOCX) [file pgen.1005562.s015.docx]

**Table S2. Plasmids used in this study.**

| **Plasmids** | **Relevant features** | **Source or reference** |
| --- | --- | --- |
| pET22b | *bla* | Novagen |
| pET28a | *kan* | Novagen |
| pRSFduet1 | *kan* | Novagen |
| pK424 | Tra^+^ Mob^+^; *bla, tet* | C. Ellermeier |
| pJS107 | TargeTron construct based on pJIR750ai (group II intron *ermB*::RAM, *ltrA*); *catP* | J. Sorg |
| pCE245 | TargeTron construct based on pJIR750ai (group II intron *ermB*::RAM, *ltrA*); *catP* | C. Ellermeier |
| pMTL83151 | pCB102, Tra^+^; *catP* | N. Minton, {Heap, 2009 #71} |
| pMTL84151 | pCD6, Tra^+^; *catP* | N. Minton, {Heap, 2009 #71} |
| pMTL84121 | pCD6, Tra^+^; *catP* | A. Henriques {Pereira, 2013 #338} |
