# Supplementary material for: Regulation of Clostridium difficile Spore Formation by the SpoIIQ and SpoIIIA Proteins
Source: PLoS Genet. 2015 Oct 14;11(10):e1005562. doi: 10.1371/journal.pgen.1005562 (PMC4605598; doi:10.1371/journal.pgen.1005562)
Supplement: S3 Table — (DOCX) [file pgen.1005562.s016.docx]

**Table S3. Primers used in this study.**

| **Primer** | **Name** | **Sequence** |
| --- | --- | --- |
| 532 | 3' Universal EBS | CGAAATTAGAAACTTGCGTTCAGTAAAC |
| 1049 | 5' IBS1.2 *spoIIIAA* 166 | AAAAAAGCTTATAATTATCCTTACAAGTCTCATAGGTGCGCCCAGATAGGGTG |
| 1050 | 3' EBS1d *spoIIIAA* 166 | CAGATTGTACAAATGTGGTGATAACAGATAAGTCTCATAGTTTAACTTACCTTTCTTTGT |
| 1051 | 5' EBS2 *spoIIIAA* 166 | TGAACGCAAGTTTCTAATTTCGGTTACTTGTCGATAGAGGAAAGTGTCT |
| 1052 | 5' IBS1.2 *spoIIQ* 456 | AAAAAAGCTTATAATTATCCTTAGAATACGGACAAGTGCGCCCAGATAGGGTG |
| 1053 | 3' EBS1d *spoIIQ* 456 | CAGATTGTACAAATGTGGTGATAACAGATAAGTCGGACAATCTAACTTACCTTTCTTTGT |
| 1054 | 5' EBS2 *spoIIQ* 456 | TGAACGCAAGTTTCTAATTTCGATTTATTCTCGATAGAGGAAAGTGTCT |
| 1074 | 5' NdeI *spoIIQ* | AGAATCATATGAAGAAAAAGCTGTTAG |
| 1075 | 3' XhoI *spoIIQ* | AATACTCGAGCTTAATTAGACTCATTGGGTC |
| 1174 | 5' NotI *spoIIIA* operon | AGAATGCGGCCGCCACTTCATGATTGAAGTGTC |
| 1175 | 3' XhoI *spoIIIA* operon | AATACTCGAGGTTTATACTTTAC |
| 1176 | 3' XhoI *spoIIIAA* | AATACTCGAGCTACTTCCTATCAAGAAAGC |
| 1177 | 5' NotI *spoIIQ* with promoter | AGAATGCGGCCGCCCACATTACTACACAGATAC |
| 1178 | 3' XhoI *spoIIQ* | AATACTCGAGGTTATACTATTACTTAATTAGACTC |
| 1239 | 3' XhoI *spoIIIAH* | AATACTCGAGCTTATTACTATTATTATTTGTAAG |
| 1264 | 5' IBS1 *spoIIIAH* 75 | AAAAAAGCTTATAATTATCCTTACTGTTCCTACTAGTGCGCCCAGATAGGGTG |
| 1265 | 3' EBS1d *spoIIIAH* 75 | CAGATTGTACAAATGTGGTGATAACAGATAAGTCCTACTACCTAACTTACCTTTCTTTGT |
| 1266 | 5' EBS2 *spoIIIAH* 75 | TGAACGCAAGTTTCTAATTTCGATTAACAGTCGATAGAGGAAAGTGTCT |
| 1277 | 3’ XhoI *C. difficile* SNAP | AAAGCTCGAGTTAACCAGCTGGACCAAGACC |
| 1301 | 5’ NheI *spoIIIAH* | TATGGCTAGCATGAAGTTTAATTATAAGGG |
| 1302 | 5' NheI *spoIIIAA* | TATGGCTAGCATGAATAAACTTTCTGATGA |
| 1303 | 3' XhoI *spoIIIAA* no stop | AATACTCGAGATTTGCAAAATACCATCTCTTTTC |
| 1313 | 3' XhoI *spoVT* | AATACTCGAGTTATTGAACTTGTTTTCC |
| 1431 | 5' *spoIIIAA* K167A SOE | CAATGTGGAGCAACAACTTTAATAAGAG |
| 1432 | 3' *spoIIIAA* K167A SOE | CTCTTATTAAAGTTGTTGCTCCACATTG |
| 1458 | 5' NdeI *cd1430* | ATACATATGAAAAAAGATAGCCTAAAAAAATACATTATGATAGG |
| 1459 | 3' XhoI *cd1430* no stop | AATCTCGAGTTTTTTATTATTTAAATAATCAAGTGTTTTAAGTTTATAACCTTC |
| 1566 | 5' NdeI *spoIIIAH* (100-691) codon opt | AGAATCATATGCTGCTGGAAACCTCAAAAGAATTC |
| 1567 | 3' XhoI *spoIIIAH* (100-691) codon opt | ACAAGCTCGAGTTTGTTTGAGTTATTGTTCG |
| 1568 | 5' NdeI *spoIIQ* (91-669) no stop codon opt | AGAATCATATGAACAATAATGTGGATAAACTG |
| 1569 | 3' XhoI *spoIIQ* (91-669) no stop codon opt | ACAAGCTCGAGTTTGATCAGGCTCATCGG |
| 1614 | 3’ XhoI *spoIIIAH* codon opt with stop | AAACTCGAGTTATTTGTTTGAGTTATTGTTCG |
| 1617 | 5' *spoIIIA* operon SOE to *spoIIIAH* | CAAAGGAGGAGGACAAGAGATGAAGTTTAATTATAAGGG |
| 1618 | 3' *spoIIIA* operon SOE to *spoIIIAH* | CCCTTATAATTAAACTTCATCTCTTGTCCTCCTCCTTTG |
| 1662 | 5' NdeI 6xHis-*spoIIQ* codon opt | AAACATATGCACCACCACCACCACCACAACAATAATGTGGATAAACTG |
| 1665 | 5’ NcoI HA-*spoIIIAH* codon opt | CCCATGGGATACCCGTACGACGTCCCGGATTATGCCCTGCTGGAAACCTCAAAAGAA |
| 1691 | 5' NcoI HA-*spoVT* | CCCATGGGATACCCGTACGACGTCCCGGACTACGCGAAAGCAACAGGTATAGTTAG |
| 1708 | 5' NdeI *spoVAD* | AATCATATGAAAAATAAAAGAATTGGAAAAAGAACAGTC |
| 1709 | 3' XhoI *spoVAD* no stop | AATCTCGAGCTCATTTACTATTACTACTGCATG |
|  |  |  |
|  |  |  |
